# Crystal structure of {(*R*)-*N* ^2^-[(benzo[*h*]quinolin-2-yl)meth­yl]-*N* ^2′^-[(benzo[*h*]quinolin-2-yl)methyl­idene]-1,1′-binaphthyl-2,2′-di­amine-κ^4^ *N*,*N*′,*N*′′,*N*′′′}(trifluoromethane­sulfonato-κ*O*)zinc(II)} trifluoromethane­sulfonate di­chloro­methane 1.5-solvate

**DOI:** 10.1107/S2056989017008027

**Published:** 2017-06-02

**Authors:** Shayna R. Skokan, Monica M. Reeson, Kayode D. Oshin, Anastasiya I. Vinokur, John A. Desper, Christopher J. Levy

**Affiliations:** a Department of Chemistry, Creighton University, Omaha, NE 68102, USA; b Department of Chemistry, University of Wisconsin, Madison, WI 53558, USA; c Department of Chemistry, Kansas State University, Manhattan, KS 66506, USA

**Keywords:** crystal structure, five-coordinate zinc(II) complex, chiral ligand, asymmetric catalysis, monohelical structure

## Abstract

The zinc(II) atom in the title compound, [Zn(C_48_H_31_N_4_)(CF_3_SO_3_)](CF_3_SO_3_)·1.5CH_2_Cl_2_, adopts a distorted five-coordinate square-pyramidal geometry. It is coordinated by one tri­fluoro­methane­sulfonate ligand and four N atoms of the *N* ^2^-[(benzo[*h*]quinolin-2-yl)meth­yl]-*N* ^2′^-[(benzo[*h*]quinolin-2-yl)methyl­idene]-1,1′-binaphthyl-2,2′-di­amine ligand. The complex is present as a single-stranded *P*-helimer monohelical structure incorporating π–π and/or σ–π inter­actions. One of the imine bonds present in the original ligand framework is reduced, leading to variations in bond lengths and torsion angles for each side of the ligand motif. The imine-bond reduction also affects the bond lengths involving the metal atom with the N-donor atoms located on the imine bond. There are two mol­ecules of the complex in the asymmetric unit. One of the mol­ecules exhibits positional disorder within the coordinating tri­fluoro­methane­sulfonate ion making the mol­ecules symmetric­ally non-equivalent.

## Abstract 2

In the title compound, the zinc(II) atom exhibits a a distorted five-coordinate square-pyramidal geometry and is coordinated by one tri­fluoro­methane­sulfonate ligand and four N-donor atoms. The resulting complex shows a single-stranded *P*-helimer structure incorporating π–π and/or σ–π inter­actions.

## Chemical context   

Stereochemistry plays a very important role in the chemical inter­actions that dominate several fields of chemistry (North, 1998 ▸). For example, in pharmacology enanti­omers of chiral drugs exhibit marked differences in toxicology, metabolism, immune response, and pharmacokinetics (Nguyen *et al.*, 2006 ▸). As a result, there is increased demand to design practical methods to synthesize monohelical chiral compounds for use as catalysts (Aspinall, 2002 ▸). Many factors contribute to the efficiency of a catalyst such as the type of metal employed, the presence of electron-donating or withdrawing functional groups, the number of chiral centers present, and regeneration capabilities (Amendola *et al.*, 1999 ▸). In addition, substrate accessibility to the metal atom plays an important role in catalytic reactions (French, 2007 ▸). Using bulky ligands in catalyst design may result in steric hindrance of the active site, a reduction in enanti­omeric excess values, and lower yields (French, 2007 ▸). Studies of catalytic mechanisms show that substrates generally approach the active site through the least hindered quadrant during a reaction (French, 2007 ▸). Designing catalysts with increased flexibility which undergo slight conformation changes as substrates approach should result in increased efficiency. This concept can be observed in nature where some enzymes can adopt flexible active sites, unlike the typical ‘lock and key’ model commonly used, allowing them to shape those active sites to accommodate bulkier substrates leading to improving efficiency (Tsou, 1993 ▸). Given the significance and application of flexible single-stranded monohelical complexes in asymmetric catalysis, we report on the synthesis and crystal structure of the solvated title compound, [Zn(C_48_H_32_N_4_)(CF_3_O_3_S)](CF_3_O_3_S)·1.5CH_2_Cl_2_ (**1**). 
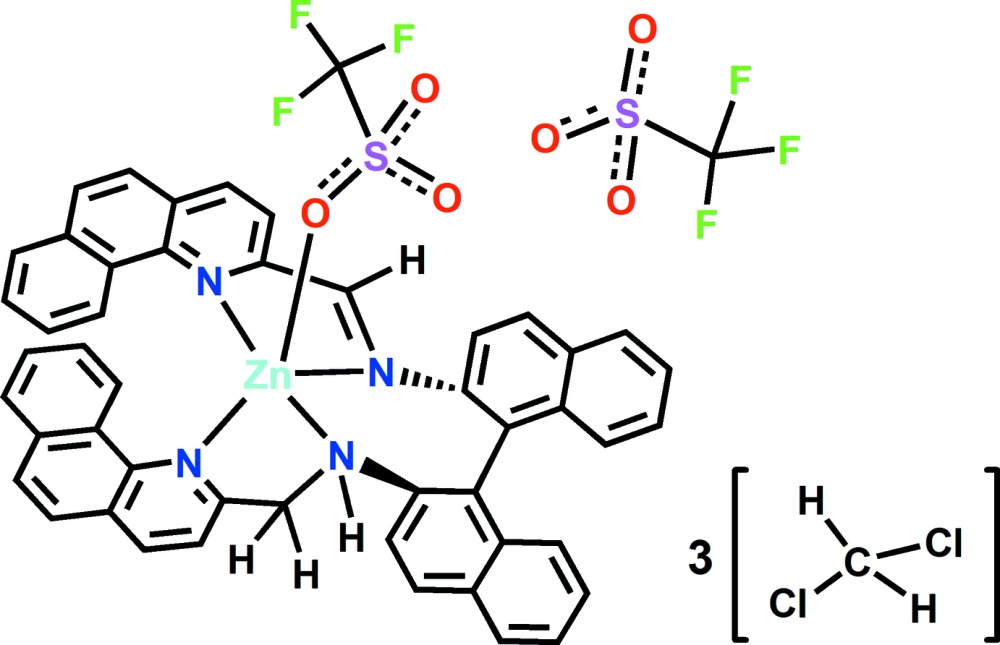


## Structural commentary   

**Figure 1:**
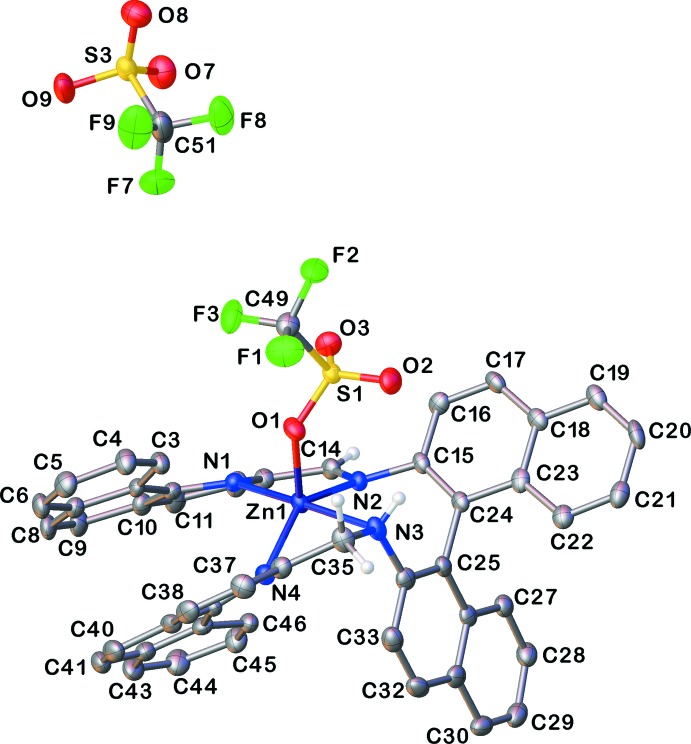
The mol­ecular structure for one of the mol­ecules of complex (**1**). Atomic displacement ellipsoids are depicted at the 50% probability level and H atoms are shown as spheres of arbitrary radius. All non-imine/amino H atoms and solvent mol­ecules have been omitted for clarity.

X-ray analysis revealed a monohelical structure (Fig. 1 ▸) with π–π and/or σ–π inter­actions between the locked side-arms of complex (**1**). The Zn^II^ cation is coordinated by four N-donor atoms from the *N* ^2^-[(benzo[*h*]quinolin-2-yl)meth­yl]-*N* ^2′^-[(benzo[*h*]quinolin-2-yl)methyl­idene]-1,1′-binaphthyl-2,2′-di­amine (BQMB) ligand and one triflate anion in a distorted square-pyramidal geometry (τ_5_ = 0.49; Addison *et al.*, 1984 ▸). We observed the reduction of one imine double bond as the C—N bond length of the unreduced imine is 1.281 (6) Å while the C—N bond length of the reduced imine is 1.433 (6) Å. We also observed the effect of the reduction in the torsion angle as the amine side (C33—C34—N3—C36) is −22.6 (5)° while the torsion angle for the imine side (C16—C15—N2—C13) is 33.0 (7)°. The reduction of the imine bond also affects the bond lengths of the zinc metal center with the N-donor atoms on the imine bond. As a result of the flexibility of the amine side, we observe a longer bond length for the Zn—N bond [2.253 (4) Å] compared to a shorter Zn—N bond length with the more rigid imine nitro­gen atom [2.056 (4) Å]. The bi­naphthalene backbone displays a twist to a degree of 76.54 (6)°.

## Supra­molecular features   

**Figure 2:**
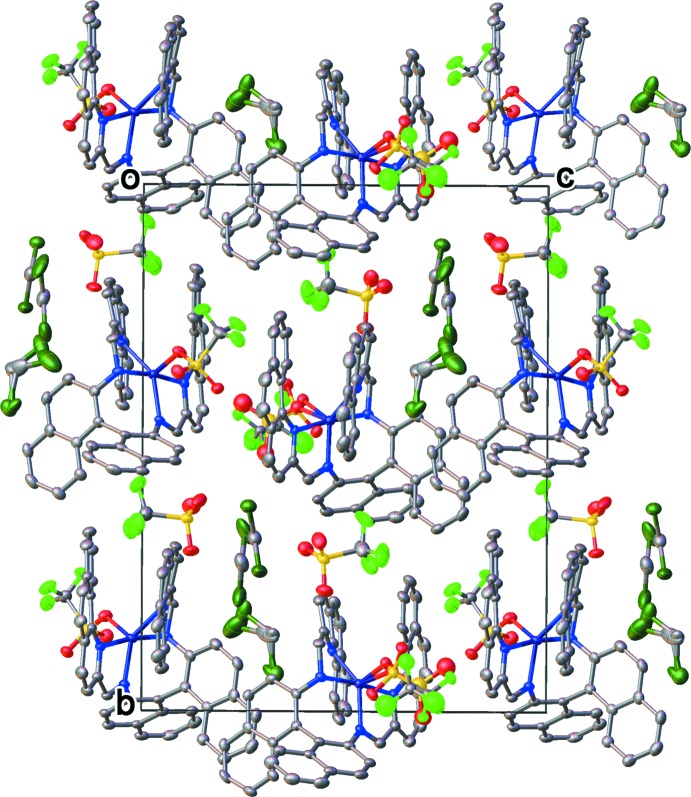
Packing diagram for complex (**1**), viewed along the *a*-axis direction. Minimal inter­actions are observed between the packed mol­ecules.

**Figure 3:**
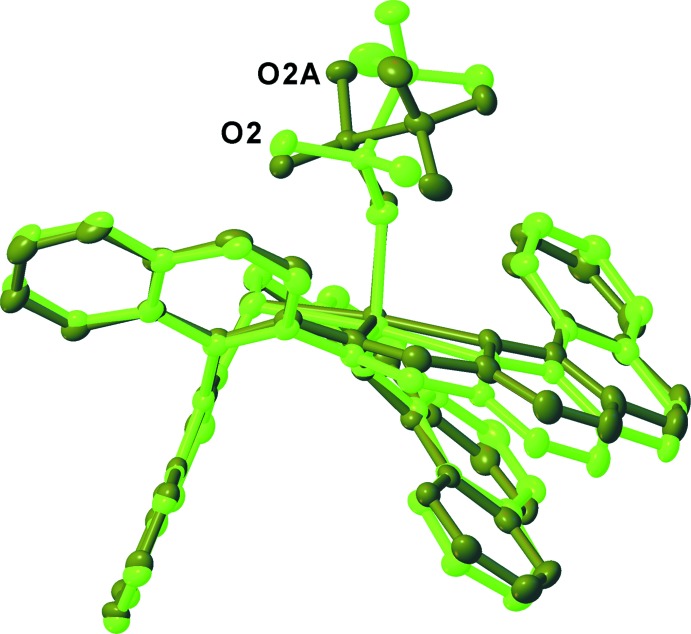
Overlay of the two mol­ecules of complex (**1**) in the asymmetric unit. Atomic displacement ellipsoids are depicted at the 50% probability and H atoms shown as spheres of arbitrary radius. All hydrogen atoms, counter-ions, solvent mol­ecules, and minor-disorder components have been omitted for clarity.

The mol­ecules of the crystal structure are related only by a twofold screw axis running along the *b*-axis direction (Fig. 2 ▸). The resulting space group *P*2_1_ is chiral. The Flack *x* and the Hooft *y* parameters were determined to be −0.008 (4) and 0.003 (4), respectively, indicating that the absolute structure was unequivocally established. Anomalous dispersion was used to determine the absolute structure. There are two mol­ecules of complex (**1**) in the asymmetric unit. As seen in Fig. 3 ▸, the difference in the two mol­ecules arises due to the orientation of the coordinating triflate. In addition, one of the mol­ecules exhibits positional disorder within the coordinating triflate ion. As a result, the two mol­ecules are not symmetry equivalent. Minimal intra­molecular inter­actions are observed between the mol­ecules of (**1**). The two mol­ecules of (**1**) in the asymmetric unit propagate along the *b*-axis direction *via* the twofold screw axis. The counter-ions and the solvent mol­ecules fill the void spaces between symmetry-related asymmetric units.

## Database survey   

The survey of Cambridge Structural Database (Groom *et al.*, 2016 ▸) revealed five instances of five-coordinate Zn complexes bonding through four amine groups and one triflate. Of the five complexes, two assume a trigonal–bipyramidal geometry (with τ_5_ values of 0.86 and 0.93), two structures have a square-pyramidal geometry (τ_5_ values of 0.02 and 0.11), and the last structure assumes a distorted square-pyramidal geometry as evidenced by the τ_5_ value of 0.48. The Zn­—O bond length for (**1**) falls on the shorter end of the distance spectrum. Meanwhile the Zn—N distances for three of the contacts agree well with those in the previously reported structures. The fourth contact at a distance of 2.253 (4) Å falls above the average Zn—N distance by 0.176 Å, presumably due to the greater flexibility within the ligand framework resulting from the imine reduction.

## Synthesis and crystallization   

**Figure 4:**
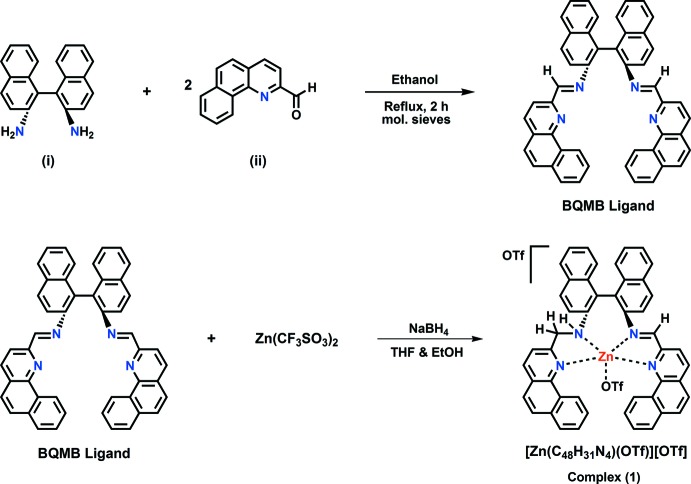
Synthetic scheme for complex (**1**)

The synthetic scheme for (**1**) is given in Fig. 4 ▸.

**Synthesis of (** ***R*** **)-** ***N***,***N*** **′-bis­[(2-benzo[** ***h*** **]quinolin­yl)methylene][1,1′-bi­naphthalene]-2,2′-di­amine (BQMB) ligand:** the BQMB ligand was synthesized following established literature procedures (Prema *et al.*, 2012 ▸). In a 100 ml round-bottom flask, (*R*)-[1,1′-binapthalene]-2,2′-di­amine (0.52 g, 1.8 mmol) and 2-formyl­benzo­quinoline (0.75 g, 3.6 mmol) were refluxed in ethanol (25 ml) for 2 h. A yellow precipitate was obtained, which was filtered and washed twice with 10 ml aliquots of ethanol. The resulting yellow mixture was dried under vacuum for 30 minutes to afford BQMB as a yellow solid (1.11 g, 92% yield). ^1^H NMR (CD_2_Cl_2_, 800 MHz): 7.32 (*t*, 2 H, *J* = 8.00 Hz, CH), 7.35 (*d*, 2 H, *J* = 8.06 Hz, CH), 7.40 (*t*, 2 H, *J* = 7.05 Hz, CH), 7.46 (*t*, 2 H, *J* = 7.00 Hz, CH), 7.55 (*t*, 2 H, *J* = 7.00 Hz, CH), 7.58 (*d*, 2 H, *J* = 8.56 Hz, CH), 7.63 (*d*, 2 H, *J* = 9.06 Hz, CH), 7.72 (*d*, 2 H, *J* = 8.56 Hz, CH), 7.77 (*d*, 2 H, *J* = 7.55 Hz, CH), 7.81 (*d*, 2 H, *J* = 8.06 Hz, CH), 8.01 (*d*, 2 H, *J* = 8.06 Hz, CH), 8.07 (*d*, 2 H, *J* = 8.06 Hz, CH), 8.10 (*d*, 2 H, *J* = 8.56 Hz, CH), 8.70 (*s*, 2 H, CH), 8.80 (*d*, 2 H, *J* = 8.06 Hz, CH). ^13^C NMR (CD_2_Cl_2_, 200 MHz): δ 119.16, 119.33, 124.39, 125.54, 125.76, 127.05, 127.35, 127.46, 127.62, 128.19, 128.34, 128.56, 128.67, 129.23, 130.04, 131.68, 132.76, 134.01, 134.04, 136.67, 146.35, 148.72, 153.89, 162.82. Elemental analysis for (C_48_H_30_N_4_): calculated C 86.98, H 4.56, N 8.45; found C 86.97, H 4.85, N 8.45.

**Synthesis of {(** ***R*** **)-** ***N*** **^2^-[(benzo[** ***h*** **]quinolin-2-yl)meth­yl]-** ***N*** **^2′^-[(benzo[** ***h*** **]quinolin-2-yl)methyl­idene]-1,1′-binaphthyl-2,2′-di­amine-κ^4^** ***N***,***N*** **′**,***N*** **′′**,***N*** **′′′}(trifluoromethane­sulfonato-κ** ***O*** **)zinc(II)} trifluoromethane­sulfonate di­chloro­methane 1.5-solvate:** the BQMB ligand (0.100 g, 0.151 mmol) was dissolved in a mixture of 15 ml ethanol and 10 ml tetra­hydro­furan in a 100 ml round-bottom flask. Sodium borohydride (0.010 g, 0.23 mmol) and zinc(II) trifluoromethanesulfonate (0.055 g, 0.15 mmol) were added to the flask to give an orange-colored solution. The reaction was allowed to reflux for 15 h and then cooled, producing a reddish-orange-colored precipitate which was filtered and washed twice with a cold solvent mixture. The precipitate was dried under vacuum for 30 minutes to yield a reddish-orange-colored solid (0.112 g, 72%). Reddish-orange-colored single crystals suitable for X-ray analysis were obtained by slow solvent diffusion of hexane into a concentrated complex solution in di­chloro­methane.

## Refinement   

Crystal data, data collection and structure refinement details are summarized in Table 1 ▸. Direct methods were used for identify positions of most of the non-hydrogen atoms and a procedure of alternating rounds between least-squares cycles and difference-Fourier maps, located the missing non-hydrogen atoms. All hydrogen atoms, except for the amine hydrogens bonded to N3 and N3*A* were refined at idealized positions and allowed to ride on neighboring atoms with relative isotropic displacement parameters. The amine hydrogen atoms were refined as riding freely. The asymmetric unit contains two mol­ecules of (**1**), two triflate counter-ions, and three mol­ecules of di­chloro­methane solvent. One of the mol­ecules of (**1**) exhibited positional disorder within the coordinating triflate ion. The positional disorder was modeled over two positions with the major component contributing 88.1 (4)%. Due to the low occupancy of the minor component, idealized geometry was used to stabilize the refinement and the component was refined isotropically. Additional disorder was observed in one of the solvent mol­ecules of di­chloro­methane. Two positions were used to model the positional disorder and the major component refined to occupancy of 50 (4)%. The bond lengths C54*A*—Cl5*A* and C54*A*—Cl6*A* were restrained to be similar.

## Supplementary Material

Crystal structure: contains datablock(s) I. DOI: 10.1107/S2056989017008027/im2476sup1.cif

Structure factors: contains datablock(s) I. DOI: 10.1107/S2056989017008027/im2476Isup2.hkl

CCDC reference: 1553147

Additional supporting information:  crystallographic information; 3D view; checkCIF report

## Figures and Tables

**Table 1:** Experimental details

Crystal data	
Chemical formula	[Zn(C_48_H_32_N_4_)(CF_3_O_3_S)](CF_3_O_3_S)·1.5CH_2_Cl_2_
*M*_r_	1155.70
Crystal system, space group	Monoclinic, *P*2_1_
Temperature (K)	120
*a*, *b*, *c* (Å)	11.837 (4), 23.126 (7), 17.836 (5)
β (°)	94.165 (10)
*V* (Å^3^)	4870 (3)
*Z*	4
Radiation type	Mo *K*α
μ (mm^−1^)	0.83
Crystal size (mm)	0.24 × 0.2 × 0.16

Data collection	
Diffractometer	Bruker APEXII CCD
Absorption correction	Multi-scan (*SADABS*; Bruker, 2016 ▸)
*T*_min_, *T*_max_	0.691, 0.744
No. of measured, independent and observed [*I* > 2σ(*I*)] reflections	100143, 19958, 17492
*R*_int_	0.073
(sin θ/λ)_max_ (Å^−1^)	0.627

Refinement	
*R*[*F* ^2^ > 2σ(*F* ^2^)], *wR*(*F* ^2^), *S*	0.039, 0.092, 1.03
No. of reflections	19958
No. of parameters	1373
No. of restraints	2
H-atom treatment	H atoms treated by a mixture of independent and constrained refinement
Δρ_max_, Δρ_min_ (e Å^−3^)	0.61, −0.46
Absolute structure	Flack *x* determined using 7334 quotients [(*I* ^+^)−(*I* ^−^)]/[(*I* ^+^)+(*I* ^−^)] (Parsons *et al*., 2013 ▸)
Absolute structure parameter	−0.008 (4)

Computer programs: *APEX2* and *SAINT* (Bruker, 2016 ▸), *SHELXT* (Sheldrick, 2015*a* ▸), *SHELXL* (Sheldrick, 2015*b* ▸) and *OLEX2* (Dolomanov *et al.*, 2009 ▸).

## supplementary crystallographic information

### Crystal data

**Table:**

[Zn(C_48_H_32_N_4_)(CF_3_O_3_S)](CF_3_O_3_S)·1.5CH_2_Cl_2_	*F*(000) = 2348
*M**_r_* = 1155.70	*D*_x_ = 1.576 Mg m^−^^3^
Monoclinic, *P*2_1_	Mo *K*α radiation, λ = 0.71073 Å
*a* = 11.837 (4) Å	Cell parameters from 9757 reflections
*b* = 23.126 (7) Å	θ = 2.2–25.8°
*c* = 17.836 (5) Å	µ = 0.83 mm^−^^1^
β = 94.165 (10)°	*T* = 120 K
*V* = 4870 (3) Å^3^	Prism, orange
*Z* = 4	0.24 × 0.2 × 0.16 mm

### Data collection

**Table:**

Bruker APEXII CCD diffractometer	19958 independent reflections
Radiation source: fine-focus sealed tube	17492 reflections with *I* > 2σ(*I*)
Graphite monochromator	*R*_int_ = 0.073
φ and ω scans	θ_max_ = 26.5°, θ_min_ = 1.2°
Absorption correction: multi-scan (SADABS; Bruker, 2016)	*h* = −14→14
*T*_min_ = 0.691, *T*_max_ = 0.744	*k* = −28→28
100143 measured reflections	*l* = −22→22

### Refinement

**Table:**

Refinement on *F*^2^	Hydrogen site location: mixed
Least-squares matrix: full	H atoms treated by a mixture of independent and constrained refinement
*R*[*F*^2^ > 2σ(*F*^2^)] = 0.039	*w* = 1/[σ^2^(*F*_o_^2^) + (0.0435*P*)^2^ + 1.1811*P*] where *P* = (*F*_o_^2^ + 2*F*_c_^2^)/3
*wR*(*F*^2^) = 0.092	(Δ/σ)_max_ < 0.001
*S* = 1.03	Δρ_max_ = 0.61 e Å^−^^3^
19958 reflections	Δρ_min_ = −0.46 e Å^−^^3^
1373 parameters	Absolute structure: Flack *x* determined using 7334 quotients [(*I*^+^)-(*I*^-^)]/[(*I*^+^)+(*I*^-^)] (Parsons et al., 2013)
2 restraints	Absolute structure parameter: −0.008 (4)
Primary atom site location: dual	

### Special details

**Table:**

Geometry. All esds (except the esd in the dihedral angle between two l.s. planes) are estimated using the full covariance matrix. The cell esds are taken into account individually in the estimation of esds in distances, angles and torsion angles; correlations between esds in cell parameters are only used when they are defined by crystal symmetry. An approximate (isotropic) treatment of cell esds is used for estimating esds involving l.s. planes.

### Fractional atomic coordinates and isotropic or equivalent isotropic displacement parameters (Å^2^)

**Table:**

	*x*	*y*	*z*	*U*_iso_*/*U*_eq_	Occ. (<1)
Zn1	0.43047 (4)	0.36244 (2)	1.02612 (3)	0.01811 (12)	
S1	0.64940 (9)	0.34588 (5)	1.13414 (6)	0.0201 (2)	
F1	0.7184 (3)	0.23936 (13)	1.14727 (19)	0.0488 (9)	
F2	0.7876 (2)	0.29519 (14)	1.23511 (17)	0.0387 (7)	
F3	0.6145 (3)	0.26621 (14)	1.23408 (19)	0.0430 (8)	
O1	0.5515 (3)	0.31988 (14)	1.09083 (19)	0.0281 (8)	
O2	0.7407 (3)	0.35829 (17)	1.08853 (19)	0.0328 (8)	
O3	0.6167 (3)	0.38875 (14)	1.18579 (18)	0.0302 (8)	
N1	0.3008 (3)	0.37869 (16)	1.1026 (2)	0.0191 (8)	
N2	0.4550 (3)	0.44962 (16)	1.04307 (19)	0.0185 (7)	
N3	0.5612 (3)	0.35750 (17)	0.9406 (2)	0.0220 (8)	
H3	0.620 (4)	0.370 (2)	0.962 (3)	0.026*	
N4	0.3657 (3)	0.30238 (16)	0.9493 (2)	0.0210 (8)	
C1	0.2150 (4)	0.3443 (2)	1.1236 (2)	0.0197 (9)	
C2	0.2297 (4)	0.28197 (19)	1.1269 (2)	0.0188 (9)	
C3	0.3357 (4)	0.2555 (2)	1.1252 (3)	0.0245 (10)	
H3B	0.401441	0.278844	1.123002	0.029*	
C4	0.3467 (4)	0.1964 (2)	1.1265 (3)	0.0299 (11)	
H4	0.419311	0.179251	1.124331	0.036*	
C5	0.2510 (4)	0.1613 (2)	1.1312 (3)	0.0307 (11)	
H5	0.257936	0.120339	1.130360	0.037*	
C6	0.1469 (4)	0.1866 (2)	1.1369 (3)	0.0283 (11)	
H6	0.082595	0.162660	1.141813	0.034*	
C7	0.1334 (4)	0.2468 (2)	1.1357 (2)	0.0231 (10)	
C8	0.0258 (4)	0.2736 (2)	1.1455 (3)	0.0275 (11)	
H8	−0.039418	0.249924	1.147935	0.033*	
C9	0.0149 (4)	0.3310 (2)	1.1513 (3)	0.0279 (11)	
H9	−0.055999	0.347123	1.161802	0.033*	
C10	0.1100 (4)	0.3686 (2)	1.1416 (2)	0.0232 (10)	
C11	0.1017 (4)	0.4291 (2)	1.1479 (3)	0.0294 (11)	
H11	0.033169	0.446261	1.161458	0.035*	
C12	0.1930 (4)	0.4635 (2)	1.1343 (3)	0.0250 (10)	
H12	0.191102	0.504075	1.142214	0.030*	
C13	0.2883 (4)	0.4367 (2)	1.1087 (2)	0.0206 (10)	
C14	0.3840 (4)	0.4718 (2)	1.0856 (3)	0.0213 (10)	
H14	0.392786	0.510679	1.102308	0.026*	
C15	0.5583 (4)	0.47927 (18)	1.0293 (3)	0.0185 (9)	
C16	0.6218 (4)	0.50475 (19)	1.0905 (3)	0.0223 (10)	
H16	0.591309	0.506703	1.138259	0.027*	
C17	0.7265 (4)	0.52656 (19)	1.0813 (3)	0.0231 (10)	
H17	0.769302	0.542936	1.123130	0.028*	
C18	0.7727 (4)	0.52526 (19)	1.0105 (3)	0.0216 (10)	
C19	0.8835 (4)	0.5465 (2)	1.0002 (3)	0.0257 (10)	
H19	0.928359	0.561884	1.041806	0.031*	
C20	0.9259 (4)	0.5450 (2)	0.9310 (3)	0.0291 (11)	
H20	0.998916	0.560544	0.924649	0.035*	
C21	0.8624 (4)	0.5207 (2)	0.8691 (3)	0.0282 (11)	
H21	0.893443	0.519083	0.821452	0.034*	
C22	0.7555 (4)	0.4994 (2)	0.8773 (3)	0.0245 (10)	
H22	0.713279	0.483253	0.835052	0.029*	
C23	0.7072 (4)	0.50106 (19)	0.9479 (3)	0.0218 (10)	
C24	0.5969 (4)	0.47783 (18)	0.9588 (2)	0.0184 (9)	
C25	0.5274 (3)	0.4541 (2)	0.8923 (2)	0.0203 (9)	
C26	0.4747 (3)	0.4929 (2)	0.8380 (2)	0.0202 (9)	
C27	0.4758 (4)	0.5536 (2)	0.8479 (3)	0.0243 (10)	
H27	0.512991	0.569823	0.891941	0.029*	
C28	0.4236 (4)	0.5895 (2)	0.7944 (3)	0.0290 (11)	
H28	0.422996	0.630107	0.802696	0.035*	
C29	0.3715 (4)	0.5667 (2)	0.7278 (3)	0.0326 (12)	
H29	0.339228	0.591900	0.690046	0.039*	
C30	0.3669 (4)	0.5083 (2)	0.7171 (3)	0.0288 (11)	
H30	0.329484	0.493049	0.672472	0.035*	
C31	0.4175 (4)	0.4701 (2)	0.7722 (3)	0.0257 (11)	
C32	0.4100 (4)	0.4093 (2)	0.7635 (3)	0.0278 (11)	
H32	0.371349	0.393618	0.719559	0.033*	
C33	0.4575 (4)	0.3728 (2)	0.8172 (3)	0.0290 (11)	
H33	0.449915	0.332199	0.810890	0.035*	
C34	0.5173 (4)	0.3951 (2)	0.8816 (2)	0.0219 (10)	
C35	0.5659 (4)	0.2948 (2)	0.9268 (3)	0.0252 (10)	
H35C	0.619432	0.276569	0.964982	0.030*	
H35D	0.593898	0.287668	0.876677	0.030*	
C36	0.4498 (4)	0.2679 (2)	0.9304 (3)	0.0230 (10)	
C37	0.4318 (4)	0.2093 (2)	0.9137 (3)	0.0280 (11)	
H37	0.492464	0.185472	0.900241	0.034*	
C38	0.3253 (4)	0.1870 (2)	0.9173 (3)	0.0285 (11)	
H38	0.313062	0.146723	0.909821	0.034*	
C39	0.2335 (4)	0.2231 (2)	0.9319 (2)	0.0251 (10)	
C40	0.1194 (4)	0.2022 (2)	0.9355 (3)	0.0313 (12)	
H40	0.103969	0.162115	0.928979	0.038*	
C41	0.0348 (4)	0.2387 (2)	0.9481 (3)	0.0305 (11)	
H41	−0.038915	0.223787	0.953597	0.037*	
C42	0.0537 (4)	0.3001 (2)	0.9532 (3)	0.0271 (11)	
C43	−0.0380 (4)	0.3386 (2)	0.9599 (3)	0.0332 (12)	
H43	−0.111839	0.323851	0.965219	0.040*	
C44	−0.0203 (4)	0.3968 (2)	0.9586 (3)	0.0335 (12)	
H44	−0.082185	0.422275	0.963780	0.040*	
C45	0.0879 (4)	0.4197 (2)	0.9497 (3)	0.0291 (11)	
H45	0.098582	0.460381	0.948053	0.035*	
C46	0.1785 (4)	0.3830 (2)	0.9434 (3)	0.0236 (10)	
H46	0.251154	0.398420	0.935725	0.028*	
C47	0.1634 (4)	0.3225 (2)	0.9484 (2)	0.0222 (10)	
C48	0.2560 (4)	0.2821 (2)	0.9441 (2)	0.0206 (9)	
C49	0.6944 (4)	0.2830 (2)	1.1909 (3)	0.0289 (11)	
Zn1A	0.02835 (4)	0.44769 (2)	0.46179 (3)	0.02053 (12)	
S1A	−0.21493 (10)	0.42631 (6)	0.37632 (7)	0.0202 (4)	0.881 (4)
S1B	−0.1362 (10)	0.4504 (6)	0.3140 (7)	0.051 (4)*	0.119 (4)
O2B	−0.1035 (17)	0.5099 (7)	0.3037 (15)	0.06 (3)*	0.119 (4)
O3B	−0.1420 (17)	0.4151 (11)	0.2470 (10)	0.07 (2)*	0.119 (4)
O1B	−0.0874 (14)	0.4228 (11)	0.3815 (11)	0.033 (16)*	0.119 (4)
C49B	−0.2872 (10)	0.4572 (8)	0.3335 (10)	0.025 (9)*	0.119 (4)
F3B	−0.3344 (14)	0.4057 (10)	0.3449 (14)	0.052 (9)*	0.119 (4)
F1B	−0.3005 (18)	0.4892 (12)	0.3948 (14)	0.093 (14)*	0.119 (4)
F2B	−0.3486 (14)	0.4823 (12)	0.2764 (15)	0.092 (19)*	0.119 (4)
F1A	−0.1634 (3)	0.4350 (2)	0.23597 (18)	0.0307 (8)	0.881 (4)
F2A	−0.2972 (3)	0.49270 (15)	0.26654 (19)	0.0329 (8)	0.881 (4)
F3A	−0.1221 (6)	0.51012 (15)	0.3023 (2)	0.034 (2)	0.881 (4)
O1A	−0.0970 (3)	0.4083 (3)	0.3952 (3)	0.0241 (10)	0.881 (4)
O2A	−0.2888 (3)	0.37945 (17)	0.3551 (2)	0.0284 (9)	0.881 (4)
O3A	−0.2536 (3)	0.46808 (16)	0.4291 (2)	0.0246 (9)	0.881 (4)
N1A	0.1442 (3)	0.46738 (17)	0.3768 (2)	0.0229 (8)	
N2A	0.0042 (3)	0.53509 (17)	0.4579 (2)	0.0230 (8)	
N3A	−0.0818 (3)	0.43485 (16)	0.5559 (2)	0.0206 (8)	
H3A	−0.144 (4)	0.449 (2)	0.541 (3)	0.025*	
N4A	0.1127 (3)	0.38621 (16)	0.5288 (2)	0.0198 (8)	
C1A	0.2263 (4)	0.4357 (2)	0.3459 (2)	0.0242 (10)	
C2A	0.2189 (4)	0.3726 (2)	0.3428 (2)	0.0230 (10)	
C3A	0.1199 (4)	0.3420 (2)	0.3550 (3)	0.0268 (11)	
H3AA	0.053265	0.362686	0.364752	0.032*	
C4A	0.1170 (5)	0.2822 (2)	0.3531 (3)	0.0314 (12)	
H4A	0.048967	0.262296	0.361758	0.038*	
C5A	0.2147 (5)	0.2511 (2)	0.3385 (3)	0.0342 (12)	
H5A	0.213924	0.210059	0.339004	0.041*	
C6A	0.3113 (5)	0.2802 (2)	0.3235 (3)	0.0342 (13)	
H6A	0.376637	0.258836	0.312619	0.041*	
C7A	0.3162 (4)	0.3412 (2)	0.3239 (3)	0.0317 (12)	
C8A	0.4150 (4)	0.3714 (3)	0.3031 (3)	0.0371 (13)	
H8A	0.480820	0.349995	0.293174	0.044*	
C9A	0.4164 (5)	0.4295 (3)	0.2973 (3)	0.0408 (14)	
H9A	0.480501	0.448180	0.279275	0.049*	
C10A	0.3222 (5)	0.4634 (2)	0.3181 (3)	0.0319 (12)	
C11A	0.3224 (5)	0.5238 (3)	0.3122 (3)	0.0375 (13)	
H11A	0.383216	0.542956	0.290575	0.045*	
C12A	0.2350 (5)	0.5550 (2)	0.3374 (3)	0.0338 (12)	
H12A	0.231904	0.595792	0.331000	0.041*	
C13A	0.1504 (4)	0.5260 (2)	0.3729 (3)	0.0261 (11)	
C14A	0.0658 (4)	0.5595 (2)	0.4099 (3)	0.0261 (10)	
H14A	0.056059	0.599449	0.398654	0.031*	
C15A	−0.0902 (4)	0.56502 (19)	0.4856 (3)	0.0219 (10)	
C16A	−0.1630 (4)	0.59740 (19)	0.4338 (3)	0.0266 (11)	
H16A	−0.142896	0.602808	0.383712	0.032*	
C17A	−0.2602 (4)	0.6203 (2)	0.4557 (3)	0.0286 (11)	
H17A	−0.307877	0.641403	0.420311	0.034*	
C18A	−0.2928 (4)	0.6138 (2)	0.5303 (3)	0.0259 (10)	
C19A	−0.3957 (4)	0.6369 (2)	0.5535 (3)	0.0315 (12)	
H19A	−0.444840	0.657552	0.518538	0.038*	
C20A	−0.4245 (4)	0.6296 (2)	0.6254 (3)	0.0340 (12)	
H20A	−0.493161	0.645592	0.640609	0.041*	
C21A	−0.3532 (4)	0.5988 (2)	0.6769 (3)	0.0336 (12)	
H21A	−0.374328	0.593785	0.726822	0.040*	
C22A	−0.2541 (4)	0.5758 (2)	0.6567 (3)	0.0254 (10)	
H22A	−0.207171	0.554962	0.692754	0.030*	
C23A	−0.2197 (4)	0.58237 (19)	0.5825 (3)	0.0217 (10)	
C24A	−0.1163 (4)	0.55782 (19)	0.5587 (3)	0.0207 (9)	
C25A	−0.0401 (4)	0.5272 (2)	0.6162 (2)	0.0193 (9)	
C26A	0.0183 (4)	0.5602 (2)	0.6746 (2)	0.0197 (9)	
C27A	0.0178 (4)	0.6223 (2)	0.6739 (3)	0.0232 (10)	
H27A	−0.021513	0.642318	0.633385	0.028*	
C28A	0.0726 (4)	0.6526 (2)	0.7302 (3)	0.0275 (11)	
H28A	0.072731	0.693640	0.728183	0.033*	
C29A	0.1296 (4)	0.6244 (2)	0.7918 (3)	0.0295 (11)	
H29A	0.165590	0.646416	0.831709	0.035*	
C30A	0.1332 (4)	0.5655 (2)	0.7943 (3)	0.0257 (11)	
H30A	0.172347	0.546685	0.835724	0.031*	
C31A	0.0788 (4)	0.5322 (2)	0.7353 (3)	0.0220 (10)	
C32A	0.0826 (4)	0.4708 (2)	0.7355 (3)	0.0228 (10)	
H32A	0.122036	0.451217	0.776327	0.027*	
C33A	0.0308 (4)	0.4395 (2)	0.6784 (2)	0.0225 (10)	
H33A	0.036188	0.398546	0.679122	0.027*	
C34A	−0.0312 (4)	0.4674 (2)	0.6179 (2)	0.0202 (9)	
C35A	−0.0815 (4)	0.37141 (19)	0.5611 (3)	0.0230 (10)	
H35A	−0.137192	0.355186	0.522608	0.028*	
H35B	−0.103773	0.359401	0.611195	0.028*	
C36A	0.0354 (4)	0.34837 (19)	0.5489 (2)	0.0224 (10)	
C37A	0.0612 (4)	0.2899 (2)	0.5614 (3)	0.0282 (11)	
H37A	0.005154	0.263667	0.576118	0.034*	
C38A	0.1693 (4)	0.2714 (2)	0.5518 (3)	0.0313 (12)	
H38A	0.187152	0.231409	0.556315	0.038*	
C39A	0.2545 (4)	0.3115 (2)	0.5353 (3)	0.0284 (11)	
C40A	0.3686 (4)	0.2939 (2)	0.5261 (3)	0.0332 (12)	
H40A	0.389013	0.254275	0.530474	0.040*	
C41A	0.4472 (5)	0.3338 (3)	0.5111 (3)	0.0379 (14)	
H41A	0.521489	0.321238	0.502162	0.045*	
C42A	0.4223 (4)	0.3936 (2)	0.5082 (3)	0.0312 (12)	
C43A	0.5066 (4)	0.4352 (3)	0.4960 (3)	0.0386 (14)	
H43A	0.580511	0.422620	0.486282	0.046*	
C44A	0.4837 (5)	0.4929 (3)	0.4979 (3)	0.0430 (15)	
H44A	0.541460	0.520091	0.489114	0.052*	
C45A	0.3751 (5)	0.5123 (3)	0.5127 (3)	0.0362 (13)	
H45A	0.360253	0.552643	0.515252	0.043*	
C46A	0.2905 (4)	0.4734 (2)	0.5236 (3)	0.0267 (11)	
H46A	0.217826	0.487037	0.534506	0.032*	
C47A	0.3097 (4)	0.4134 (2)	0.5188 (2)	0.0227 (10)	
C48A	0.2244 (3)	0.3701 (2)	0.5274 (2)	0.0226 (10)	
C49A	−0.1989 (5)	0.4679 (2)	0.2906 (3)	0.0262 (12)	0.881 (4)
Cl1	0.58159 (12)	0.61215 (6)	0.26355 (8)	0.0438 (3)	
Cl2	0.5759 (2)	0.73553 (7)	0.29120 (10)	0.0718 (6)	
C52	0.4935 (5)	0.6722 (3)	0.2787 (3)	0.0453 (14)	
H52A	0.437762	0.677108	0.234983	0.054*	
H52B	0.451332	0.665405	0.323785	0.054*	
Cl3	−0.0760 (2)	0.65839 (12)	0.25389 (12)	0.1000 (9)	
Cl4	−0.0606 (2)	0.78324 (10)	0.24111 (10)	0.0820 (7)	
C53	0.0145 (6)	0.7184 (3)	0.2463 (4)	0.0556 (17)	
H53A	0.070264	0.719394	0.290410	0.067*	
H53B	0.056829	0.714086	0.200694	0.067*	
Cl5	0.766 (2)	0.3383 (10)	0.7706 (14)	0.116 (6)	0.50 (4)
Cl6	0.6838 (15)	0.4281 (7)	0.6701 (10)	0.051 (3)	0.50 (4)
C54	0.672 (4)	0.3528 (14)	0.691 (2)	0.078 (14)	0.50 (4)
H54A	0.592794	0.343386	0.701713	0.094*	0.50 (4)
H54B	0.691946	0.329229	0.647525	0.094*	0.50 (4)
Cl5A	0.7225 (10)	0.3115 (9)	0.7495 (4)	0.076 (4)	0.50 (4)
Cl6A	0.6937 (17)	0.4266 (7)	0.6793 (10)	0.062 (4)	0.50 (4)
C54A	0.654 (3)	0.3534 (9)	0.678 (2)	0.037 (6)	0.50 (4)
H54C	0.571251	0.350914	0.682258	0.045*	0.50 (4)
H54D	0.670213	0.336652	0.628793	0.045*	0.50 (4)
S3	0.26214 (10)	0.71026 (5)	0.44855 (7)	0.0279 (3)	
F7	0.3110 (4)	0.7034 (2)	0.5944 (2)	0.0800 (14)	
F8	0.1374 (3)	0.72046 (18)	0.5629 (2)	0.0651 (11)	
F9	0.2069 (4)	0.63445 (17)	0.5491 (2)	0.0727 (13)	
O7	0.2765 (3)	0.77162 (16)	0.4520 (2)	0.0439 (10)	
O8	0.1623 (3)	0.69187 (18)	0.4047 (2)	0.0445 (10)	
O9	0.3614 (3)	0.67666 (19)	0.4381 (2)	0.0446 (10)	
C51	0.2293 (5)	0.6917 (3)	0.5441 (3)	0.0492 (16)	
S2	0.24274 (11)	0.63536 (5)	0.11069 (7)	0.0278 (3)	
F4	0.1672 (4)	0.65490 (19)	−0.0282 (2)	0.0742 (13)	
F5	0.3472 (4)	0.6473 (2)	−0.0134 (2)	0.0760 (13)	
F6	0.2426 (3)	0.57099 (16)	−0.0104 (2)	0.0601 (10)	
O4	0.2534 (4)	0.69671 (15)	0.1215 (2)	0.0440 (10)	
O5	0.3366 (3)	0.60187 (17)	0.1411 (2)	0.0480 (11)	
O6	0.1339 (3)	0.6118 (2)	0.1215 (3)	0.0539 (11)	
C50	0.2519 (5)	0.6268 (3)	0.0102 (3)	0.0416 (14)	

### Atomic displacement parameters (Å^2^)

**Table:**

	*U*^11^	*U*^22^	*U*^33^	*U*^12^	*U*^13^	*U*^23^
Zn1	0.0164 (2)	0.0137 (2)	0.0242 (3)	−0.0006 (2)	0.00162 (19)	−0.0021 (2)
S1	0.0161 (5)	0.0186 (6)	0.0254 (5)	0.0008 (4)	0.0008 (4)	0.0015 (5)
F1	0.064 (2)	0.0269 (17)	0.055 (2)	0.0211 (16)	−0.0039 (17)	−0.0070 (16)
F2	0.0262 (15)	0.048 (2)	0.0407 (17)	0.0057 (14)	−0.0051 (13)	0.0107 (15)
F3	0.0340 (17)	0.0387 (19)	0.058 (2)	0.0033 (14)	0.0110 (15)	0.0240 (16)
O1	0.0205 (16)	0.0248 (18)	0.0381 (19)	−0.0011 (14)	−0.0041 (14)	0.0048 (15)
O2	0.0213 (16)	0.040 (2)	0.0380 (19)	0.0007 (16)	0.0061 (14)	0.0058 (18)
O3	0.0378 (19)	0.0225 (18)	0.0302 (18)	0.0035 (15)	0.0026 (15)	−0.0044 (14)
N1	0.0202 (19)	0.018 (2)	0.0188 (18)	−0.0003 (15)	0.0011 (15)	0.0011 (15)
N2	0.0191 (18)	0.0145 (18)	0.0217 (17)	−0.0013 (16)	0.0008 (14)	0.0014 (16)
N3	0.0179 (18)	0.016 (2)	0.032 (2)	0.0003 (17)	0.0040 (16)	−0.0043 (18)
N4	0.024 (2)	0.016 (2)	0.0233 (19)	−0.0019 (16)	0.0003 (15)	−0.0009 (16)
C1	0.020 (2)	0.022 (2)	0.017 (2)	−0.0022 (18)	0.0003 (17)	0.0016 (18)
C2	0.022 (2)	0.016 (2)	0.018 (2)	−0.0040 (18)	0.0011 (18)	0.0033 (18)
C3	0.021 (2)	0.022 (3)	0.031 (3)	−0.0028 (19)	−0.001 (2)	0.005 (2)
C4	0.026 (3)	0.024 (3)	0.040 (3)	0.001 (2)	0.001 (2)	0.010 (2)
C5	0.035 (3)	0.020 (3)	0.037 (3)	0.000 (2)	0.002 (2)	0.006 (2)
C6	0.025 (3)	0.024 (3)	0.036 (3)	−0.008 (2)	0.003 (2)	0.005 (2)
C7	0.024 (2)	0.023 (3)	0.022 (2)	−0.003 (2)	0.0021 (19)	0.002 (2)
C8	0.020 (2)	0.031 (3)	0.033 (3)	−0.007 (2)	0.007 (2)	0.006 (2)
C9	0.021 (2)	0.032 (3)	0.032 (3)	−0.001 (2)	0.011 (2)	0.004 (2)
C10	0.022 (2)	0.026 (3)	0.023 (2)	−0.001 (2)	0.0062 (17)	0.000 (2)
C11	0.028 (3)	0.031 (3)	0.031 (3)	0.001 (2)	0.013 (2)	−0.001 (2)
C12	0.029 (3)	0.018 (2)	0.029 (2)	0.003 (2)	0.010 (2)	−0.002 (2)
C13	0.020 (2)	0.019 (2)	0.024 (2)	0.0016 (19)	0.0002 (18)	0.0015 (19)
C14	0.025 (2)	0.014 (2)	0.025 (2)	−0.0026 (19)	0.0001 (19)	0.0008 (19)
C15	0.016 (2)	0.011 (2)	0.028 (2)	0.0001 (17)	0.0002 (18)	0.0022 (18)
C16	0.029 (3)	0.013 (2)	0.025 (2)	0.0014 (19)	0.0039 (19)	−0.0006 (19)
C17	0.020 (2)	0.015 (2)	0.033 (3)	−0.0024 (19)	−0.0046 (19)	0.001 (2)
C18	0.021 (2)	0.011 (2)	0.032 (3)	0.0010 (18)	−0.0016 (19)	0.0007 (19)
C19	0.020 (2)	0.014 (2)	0.043 (3)	0.0001 (18)	−0.001 (2)	0.002 (2)
C20	0.015 (2)	0.020 (3)	0.053 (3)	−0.0034 (19)	0.004 (2)	0.008 (2)
C21	0.024 (2)	0.026 (3)	0.036 (3)	0.006 (2)	0.010 (2)	0.011 (2)
C22	0.023 (2)	0.019 (2)	0.032 (3)	0.004 (2)	0.0023 (19)	0.005 (2)
C23	0.020 (2)	0.013 (2)	0.033 (2)	0.0051 (18)	0.0053 (19)	0.0062 (19)
C24	0.018 (2)	0.011 (2)	0.026 (2)	0.0000 (17)	−0.0002 (18)	0.0022 (18)
C25	0.016 (2)	0.022 (2)	0.023 (2)	−0.0033 (19)	0.0041 (17)	−0.0002 (19)
C26	0.013 (2)	0.024 (2)	0.024 (2)	−0.0001 (19)	0.0064 (17)	−0.001 (2)
C27	0.018 (2)	0.027 (3)	0.028 (2)	−0.002 (2)	0.0035 (18)	0.002 (2)
C28	0.021 (2)	0.030 (3)	0.036 (3)	0.000 (2)	0.001 (2)	0.009 (2)
C29	0.023 (3)	0.041 (3)	0.034 (3)	0.002 (2)	0.004 (2)	0.015 (2)
C30	0.021 (2)	0.043 (3)	0.022 (2)	0.000 (2)	0.0008 (19)	0.005 (2)
C31	0.021 (2)	0.034 (3)	0.023 (2)	−0.002 (2)	0.0079 (19)	0.001 (2)
C32	0.025 (2)	0.036 (3)	0.022 (2)	−0.004 (2)	0.0018 (19)	−0.006 (2)
C33	0.030 (3)	0.027 (3)	0.031 (3)	−0.005 (2)	0.007 (2)	−0.005 (2)
C34	0.019 (2)	0.025 (3)	0.023 (2)	0.0012 (19)	0.0074 (18)	−0.0022 (19)
C35	0.026 (2)	0.016 (2)	0.034 (3)	0.0040 (19)	0.006 (2)	−0.006 (2)
C36	0.030 (3)	0.016 (2)	0.024 (2)	0.000 (2)	0.002 (2)	−0.0006 (19)
C37	0.032 (3)	0.019 (2)	0.033 (3)	0.003 (2)	0.001 (2)	−0.004 (2)
C38	0.043 (3)	0.015 (2)	0.027 (3)	−0.006 (2)	0.000 (2)	−0.003 (2)
C39	0.033 (3)	0.019 (2)	0.022 (2)	−0.007 (2)	−0.0050 (19)	0.0006 (19)
C40	0.039 (3)	0.024 (3)	0.030 (3)	−0.012 (2)	−0.005 (2)	0.002 (2)
C41	0.027 (3)	0.033 (3)	0.031 (3)	−0.011 (2)	−0.004 (2)	0.006 (2)
C42	0.027 (3)	0.032 (3)	0.021 (2)	−0.005 (2)	−0.0018 (19)	0.005 (2)
C43	0.018 (2)	0.045 (3)	0.036 (3)	−0.002 (2)	−0.001 (2)	0.012 (3)
C44	0.024 (3)	0.036 (3)	0.041 (3)	0.006 (2)	0.002 (2)	0.009 (3)
C45	0.027 (3)	0.023 (3)	0.037 (3)	0.003 (2)	0.002 (2)	0.008 (2)
C46	0.024 (2)	0.024 (3)	0.023 (2)	−0.005 (2)	−0.0020 (19)	0.0047 (19)
C47	0.024 (2)	0.023 (2)	0.019 (2)	0.001 (2)	−0.0021 (18)	0.0046 (19)
C48	0.022 (2)	0.019 (2)	0.020 (2)	−0.0044 (19)	−0.0015 (18)	0.0016 (19)
C49	0.025 (3)	0.027 (3)	0.035 (3)	0.006 (2)	0.004 (2)	0.004 (2)
Zn1A	0.0228 (3)	0.0172 (3)	0.0216 (2)	0.0004 (2)	0.0015 (2)	−0.0015 (2)
S1A	0.0182 (7)	0.0222 (7)	0.0202 (6)	−0.0023 (5)	0.0003 (5)	−0.0024 (5)
F1A	0.0333 (18)	0.036 (2)	0.0239 (16)	−0.0029 (19)	0.0075 (14)	−0.0064 (16)
F2A	0.036 (2)	0.0283 (19)	0.0333 (18)	0.0085 (17)	−0.0054 (16)	0.0015 (15)
F3A	0.043 (2)	0.030 (3)	0.028 (3)	−0.0183 (16)	0.0003 (15)	0.0001 (14)
O1A	0.020 (2)	0.027 (2)	0.025 (2)	−0.0004 (18)	−0.0012 (16)	−0.002 (2)
O2A	0.026 (2)	0.028 (2)	0.030 (2)	−0.0095 (18)	−0.0012 (16)	−0.0027 (17)
O3A	0.0201 (19)	0.028 (2)	0.0259 (19)	−0.0029 (16)	0.0045 (15)	−0.0077 (16)
N1A	0.030 (2)	0.022 (2)	0.0171 (18)	−0.0005 (17)	0.0033 (16)	−0.0027 (16)
N2A	0.028 (2)	0.019 (2)	0.0209 (19)	0.0006 (17)	−0.0020 (16)	−0.0047 (16)
N3A	0.0195 (19)	0.018 (2)	0.0243 (19)	−0.0005 (16)	0.0011 (16)	−0.0007 (16)
N4A	0.0222 (19)	0.0180 (19)	0.0193 (18)	−0.0006 (16)	0.0015 (15)	−0.0037 (15)
C1A	0.027 (2)	0.028 (3)	0.017 (2)	0.000 (2)	0.0024 (18)	−0.0004 (19)
C2A	0.026 (2)	0.023 (3)	0.020 (2)	0.003 (2)	0.0022 (18)	−0.0051 (19)
C3A	0.031 (3)	0.024 (3)	0.025 (2)	0.003 (2)	−0.003 (2)	−0.008 (2)
C4A	0.036 (3)	0.028 (3)	0.030 (3)	−0.003 (2)	−0.001 (2)	−0.010 (2)
C5A	0.045 (3)	0.025 (3)	0.033 (3)	0.006 (2)	0.002 (2)	−0.008 (2)
C6A	0.038 (3)	0.036 (3)	0.029 (3)	0.015 (3)	0.005 (2)	−0.007 (2)
C7A	0.033 (3)	0.038 (3)	0.024 (2)	0.004 (2)	0.005 (2)	−0.004 (2)
C8A	0.033 (3)	0.045 (4)	0.035 (3)	0.008 (3)	0.014 (2)	−0.006 (3)
C9A	0.034 (3)	0.051 (4)	0.040 (3)	−0.004 (3)	0.017 (2)	−0.008 (3)
C10A	0.041 (3)	0.032 (3)	0.024 (2)	−0.001 (2)	0.009 (2)	−0.003 (2)
C11A	0.039 (3)	0.042 (3)	0.034 (3)	−0.011 (3)	0.017 (2)	−0.004 (3)
C12A	0.044 (3)	0.028 (3)	0.031 (3)	−0.010 (2)	0.009 (2)	0.001 (2)
C13A	0.035 (3)	0.024 (3)	0.020 (2)	−0.005 (2)	0.003 (2)	−0.001 (2)
C14A	0.031 (3)	0.021 (2)	0.026 (2)	−0.003 (2)	0.000 (2)	0.000 (2)
C15A	0.021 (2)	0.015 (2)	0.030 (2)	−0.0022 (18)	0.0004 (19)	−0.0025 (19)
C16A	0.039 (3)	0.014 (2)	0.026 (2)	−0.001 (2)	−0.002 (2)	−0.002 (2)
C17A	0.030 (3)	0.015 (2)	0.039 (3)	−0.002 (2)	−0.009 (2)	0.001 (2)
C18A	0.026 (2)	0.014 (2)	0.037 (3)	−0.002 (2)	−0.005 (2)	−0.002 (2)
C19A	0.021 (2)	0.023 (3)	0.050 (3)	0.004 (2)	−0.004 (2)	0.005 (2)
C20A	0.024 (3)	0.025 (3)	0.054 (3)	0.001 (2)	0.009 (2)	0.000 (2)
C21A	0.024 (3)	0.036 (3)	0.042 (3)	0.001 (2)	0.009 (2)	0.000 (2)
C22A	0.022 (2)	0.019 (2)	0.035 (3)	0.0002 (19)	0.001 (2)	0.001 (2)
C23A	0.021 (2)	0.013 (2)	0.030 (2)	−0.0022 (18)	−0.0013 (19)	−0.0028 (19)
C24A	0.022 (2)	0.013 (2)	0.026 (2)	−0.0009 (18)	0.0017 (18)	−0.0019 (19)
C25A	0.017 (2)	0.019 (2)	0.022 (2)	−0.0028 (18)	0.0040 (17)	0.0024 (19)
C26A	0.018 (2)	0.022 (2)	0.021 (2)	−0.0015 (18)	0.0058 (17)	−0.0014 (19)
C27A	0.020 (2)	0.025 (3)	0.026 (2)	−0.0008 (19)	0.0055 (18)	−0.004 (2)
C28A	0.029 (3)	0.019 (2)	0.036 (3)	−0.003 (2)	0.008 (2)	−0.008 (2)
C29A	0.025 (2)	0.036 (3)	0.027 (2)	−0.005 (2)	0.000 (2)	−0.015 (2)
C30A	0.018 (2)	0.037 (3)	0.022 (2)	−0.001 (2)	0.0002 (18)	−0.001 (2)
C31A	0.016 (2)	0.027 (3)	0.023 (2)	−0.0001 (19)	0.0070 (18)	−0.003 (2)
C32A	0.018 (2)	0.027 (3)	0.024 (2)	0.0008 (19)	0.0009 (18)	0.003 (2)
C33A	0.020 (2)	0.020 (3)	0.029 (2)	0.0029 (19)	0.0076 (18)	0.003 (2)
C34A	0.020 (2)	0.021 (2)	0.021 (2)	−0.0039 (18)	0.0068 (18)	−0.0036 (18)
C35A	0.025 (2)	0.015 (2)	0.029 (2)	−0.0066 (19)	0.0028 (19)	0.000 (2)
C36A	0.027 (2)	0.019 (2)	0.021 (2)	−0.0040 (19)	−0.0018 (18)	−0.0018 (19)
C37A	0.035 (3)	0.021 (3)	0.029 (3)	−0.006 (2)	0.005 (2)	0.000 (2)
C38A	0.040 (3)	0.021 (3)	0.032 (3)	0.003 (2)	0.002 (2)	0.001 (2)
C39A	0.034 (3)	0.028 (3)	0.023 (2)	0.008 (2)	−0.001 (2)	−0.006 (2)
C40A	0.032 (3)	0.037 (3)	0.031 (3)	0.013 (2)	0.002 (2)	0.001 (2)
C41A	0.026 (3)	0.057 (4)	0.031 (3)	0.014 (3)	0.003 (2)	−0.004 (3)
C42A	0.031 (3)	0.042 (3)	0.020 (2)	0.001 (2)	−0.001 (2)	−0.004 (2)
C43A	0.021 (2)	0.056 (4)	0.039 (3)	−0.005 (3)	0.005 (2)	−0.009 (3)
C44A	0.035 (3)	0.056 (4)	0.039 (3)	−0.019 (3)	0.008 (2)	−0.014 (3)
C45A	0.041 (3)	0.038 (3)	0.031 (3)	−0.016 (3)	0.006 (2)	−0.010 (2)
C46A	0.024 (3)	0.035 (3)	0.021 (2)	−0.007 (2)	0.0039 (19)	−0.006 (2)
C47A	0.023 (2)	0.030 (3)	0.015 (2)	−0.002 (2)	0.0007 (18)	−0.0058 (19)
C48A	0.019 (2)	0.027 (3)	0.021 (2)	0.002 (2)	0.0001 (17)	−0.002 (2)
C49A	0.030 (3)	0.022 (3)	0.026 (3)	−0.001 (2)	−0.003 (2)	−0.004 (2)
Cl1	0.0474 (8)	0.0329 (7)	0.0511 (8)	0.0016 (6)	0.0038 (7)	−0.0014 (6)
Cl2	0.1357 (18)	0.0293 (8)	0.0479 (9)	−0.0008 (10)	−0.0115 (10)	−0.0003 (7)
C52	0.052 (4)	0.047 (4)	0.038 (3)	0.010 (3)	0.005 (3)	0.002 (3)
Cl3	0.1141 (18)	0.125 (2)	0.0597 (12)	−0.0453 (16)	−0.0013 (12)	0.0457 (13)
Cl4	0.1173 (17)	0.0904 (16)	0.0369 (9)	0.0320 (13)	−0.0048 (10)	−0.0103 (10)
C53	0.057 (4)	0.070 (5)	0.039 (3)	−0.004 (4)	0.000 (3)	0.012 (3)
Cl5	0.129 (10)	0.105 (9)	0.117 (8)	0.056 (8)	0.044 (9)	0.051 (7)
Cl6	0.045 (4)	0.056 (6)	0.052 (4)	−0.003 (4)	0.009 (3)	0.007 (4)
C54	0.08 (2)	0.061 (19)	0.10 (3)	−0.029 (14)	0.07 (2)	−0.027 (14)
Cl5A	0.070 (5)	0.111 (8)	0.048 (3)	0.046 (4)	0.021 (2)	0.022 (3)
Cl6A	0.069 (7)	0.056 (7)	0.063 (6)	−0.025 (5)	0.031 (5)	−0.035 (5)
C54A	0.044 (10)	0.031 (12)	0.038 (8)	0.014 (9)	0.009 (8)	0.001 (8)
S3	0.0268 (6)	0.0253 (7)	0.0321 (6)	0.0048 (5)	0.0046 (5)	0.0052 (5)
F7	0.083 (3)	0.116 (4)	0.038 (2)	0.036 (3)	−0.015 (2)	−0.008 (2)
F8	0.064 (2)	0.078 (3)	0.058 (2)	0.031 (2)	0.0326 (19)	0.017 (2)
F9	0.106 (3)	0.048 (2)	0.069 (3)	0.016 (2)	0.037 (2)	0.029 (2)
O7	0.048 (2)	0.028 (2)	0.057 (3)	−0.0015 (18)	0.009 (2)	0.0033 (19)
O8	0.044 (2)	0.040 (2)	0.048 (2)	0.0005 (19)	−0.0057 (19)	0.0010 (19)
O9	0.037 (2)	0.055 (3)	0.043 (2)	0.020 (2)	0.0119 (17)	0.003 (2)
C51	0.048 (4)	0.056 (4)	0.045 (4)	0.024 (3)	0.013 (3)	0.006 (3)
S2	0.0325 (7)	0.0196 (6)	0.0313 (6)	0.0007 (5)	0.0032 (5)	0.0001 (5)
F4	0.093 (3)	0.083 (3)	0.043 (2)	0.035 (3)	−0.017 (2)	0.001 (2)
F5	0.076 (3)	0.099 (4)	0.057 (2)	−0.019 (3)	0.034 (2)	−0.009 (2)
F6	0.075 (3)	0.052 (2)	0.051 (2)	0.012 (2)	−0.0135 (18)	−0.0237 (18)
O4	0.062 (3)	0.0195 (19)	0.051 (2)	−0.0061 (18)	0.005 (2)	−0.0048 (18)
O5	0.059 (3)	0.038 (2)	0.043 (2)	0.020 (2)	−0.0187 (19)	−0.0133 (19)
O6	0.046 (2)	0.052 (3)	0.066 (3)	−0.016 (2)	0.018 (2)	−0.002 (2)
C50	0.048 (3)	0.039 (3)	0.037 (3)	0.002 (3)	−0.001 (3)	−0.004 (3)

### Geometric parameters (Å, º)

**Table:**

Zn1—O1	2.028 (3)	F3A—C49A	1.340 (7)
Zn1—N1	2.160 (4)	N1A—C1A	1.365 (6)
Zn1—N2	2.056 (4)	N1A—C13A	1.361 (6)
Zn1—N3	2.253 (4)	N2A—C14A	1.295 (6)
Zn1—N4	2.059 (4)	N2A—C15A	1.433 (6)
S1—O1	1.473 (3)	N3A—H3A	0.83 (5)
S1—O2	1.428 (3)	N3A—C34A	1.432 (6)
S1—O3	1.426 (3)	N3A—C35A	1.470 (6)
S1—C49	1.829 (5)	N4A—C36A	1.334 (6)
F1—C49	1.318 (6)	N4A—C48A	1.376 (5)
F2—C49	1.339 (6)	C1A—C2A	1.463 (7)
F3—C49	1.321 (6)	C1A—C10A	1.424 (7)
N1—C1	1.362 (6)	C2A—C3A	1.400 (7)
N1—C13	1.354 (6)	C2A—C7A	1.422 (7)
N2—C14	1.281 (6)	C3A—H3AA	0.9500
N2—C15	1.439 (5)	C3A—C4A	1.382 (7)
N3—H3	0.82 (5)	C4A—H4A	0.9500
N3—C34	1.433 (6)	C4A—C5A	1.403 (7)
N3—C35	1.473 (6)	C5A—H5A	0.9500
N4—C36	1.337 (6)	C5A—C6A	1.369 (8)
N4—C48	1.377 (6)	C6A—H6A	0.9500
C1—C2	1.453 (6)	C6A—C7A	1.410 (8)
C1—C10	1.422 (6)	C7A—C8A	1.435 (7)
C2—C3	1.399 (6)	C8A—H8A	0.9500
C2—C7	1.418 (6)	C8A—C9A	1.348 (8)
C3—H3B	0.9500	C9A—H9A	0.9500
C3—C4	1.373 (7)	C9A—C10A	1.434 (8)
C4—H4	0.9500	C10A—C11A	1.401 (8)
C4—C5	1.402 (7)	C11A—H11A	0.9500
C5—H5	0.9500	C11A—C12A	1.365 (8)
C5—C6	1.374 (7)	C12A—H12A	0.9500
C6—H6	0.9500	C12A—C13A	1.394 (7)
C6—C7	1.403 (7)	C13A—C14A	1.460 (7)
C7—C8	1.439 (7)	C14A—H14A	0.9500
C8—H8	0.9500	C15A—C16A	1.429 (6)
C8—C9	1.338 (7)	C15A—C24A	1.370 (6)
C9—H9	0.9500	C16A—H16A	0.9500
C9—C10	1.442 (6)	C16A—C17A	1.349 (7)
C10—C11	1.408 (7)	C17A—H17A	0.9500
C11—H11	0.9500	C17A—C18A	1.420 (7)
C11—C12	1.377 (7)	C18A—C19A	1.419 (7)
C12—H12	0.9500	C18A—C23A	1.423 (6)
C12—C13	1.393 (6)	C19A—H19A	0.9500
C13—C14	1.476 (6)	C19A—C20A	1.362 (8)
C14—H14	0.9500	C20A—H20A	0.9500
C15—C16	1.408 (6)	C20A—C21A	1.397 (7)
C15—C24	1.369 (6)	C21A—H21A	0.9500
C16—H16	0.9500	C21A—C22A	1.360 (7)
C16—C17	1.360 (6)	C22A—H22A	0.9500
C17—H17	0.9500	C22A—C23A	1.421 (7)
C17—C18	1.412 (7)	C23A—C24A	1.441 (6)
C18—C19	1.425 (6)	C24A—C25A	1.495 (6)
C18—C23	1.428 (6)	C25A—C26A	1.427 (6)
C19—H19	0.9500	C25A—C34A	1.388 (6)
C19—C20	1.365 (7)	C26A—C27A	1.437 (6)
C20—H20	0.9500	C26A—C31A	1.413 (6)
C20—C21	1.407 (7)	C27A—H27A	0.9500
C21—H21	0.9500	C27A—C28A	1.352 (7)
C21—C22	1.377 (7)	C28A—H28A	0.9500
C22—H22	0.9500	C28A—C29A	1.407 (7)
C22—C23	1.420 (7)	C29A—H29A	0.9500
C23—C24	1.438 (6)	C29A—C30A	1.362 (7)
C24—C25	1.499 (6)	C30A—H30A	0.9500
C25—C26	1.430 (6)	C30A—C31A	1.420 (6)
C25—C34	1.381 (7)	C31A—C32A	1.420 (7)
C26—C27	1.414 (7)	C32A—H32A	0.9500
C26—C31	1.415 (6)	C32A—C33A	1.358 (6)
C27—H27	0.9500	C33A—H33A	0.9500
C27—C28	1.376 (7)	C33A—C34A	1.415 (6)
C28—H28	0.9500	C35A—H35A	0.9900
C28—C29	1.402 (7)	C35A—H35B	0.9900
C29—H29	0.9500	C35A—C36A	1.513 (6)
C29—C30	1.364 (7)	C36A—C37A	1.401 (7)
C30—H30	0.9500	C37A—H37A	0.9500
C30—C31	1.421 (7)	C37A—C38A	1.372 (7)
C31—C32	1.417 (7)	C38A—H38A	0.9500
C32—H32	0.9500	C38A—C39A	1.416 (7)
C32—C33	1.366 (7)	C39A—C40A	1.432 (7)
C33—H33	0.9500	C39A—C48A	1.407 (7)
C33—C34	1.403 (6)	C40A—H40A	0.9500
C35—H35C	0.9900	C40A—C41A	1.350 (8)
C35—H35D	0.9900	C41A—H41A	0.9500
C35—C36	1.514 (7)	C41A—C42A	1.416 (8)
C36—C37	1.401 (7)	C42A—C43A	1.413 (8)
C37—H37	0.9500	C42A—C47A	1.435 (7)
C37—C38	1.368 (7)	C43A—H43A	0.9500
C38—H38	0.9500	C43A—C44A	1.363 (9)
C38—C39	1.409 (7)	C44A—H44A	0.9500
C39—C40	1.440 (7)	C44A—C45A	1.405 (8)
C39—C48	1.404 (6)	C45A—H45A	0.9500
C40—H40	0.9500	C45A—C46A	1.372 (7)
C40—C41	1.342 (7)	C46A—H46A	0.9500
C41—H41	0.9500	C46A—C47A	1.410 (7)
C41—C42	1.440 (7)	C47A—C48A	1.437 (6)
C42—C43	1.415 (7)	Cl1—C52	1.770 (6)
C42—C47	1.406 (7)	Cl2—C52	1.764 (7)
C43—H43	0.9500	C52—H52A	0.9900
C43—C44	1.361 (8)	C52—H52B	0.9900
C44—H44	0.9500	Cl3—C53	1.765 (7)
C44—C45	1.406 (7)	Cl4—C53	1.743 (7)
C45—H45	0.9500	C53—H53A	0.9900
C45—C46	1.378 (7)	C53—H53B	0.9900
C46—H46	0.9500	Cl5—C54	1.77 (4)
C46—C47	1.416 (7)	Cl6—C54	1.79 (3)
C47—C48	1.447 (6)	C54—H54A	0.9900
Zn1A—O1B	1.994 (19)	C54—H54B	0.9900
Zn1A—O1A	2.045 (4)	Cl5A—C54A	1.75 (2)
Zn1A—N1A	2.165 (4)	Cl6A—C54A	1.76 (2)
Zn1A—N2A	2.042 (4)	C54A—H54C	0.9900
Zn1A—N3A	2.219 (4)	C54A—H54D	0.9900
Zn1A—N4A	2.068 (4)	S3—O7	1.430 (4)
S1A—O1A	1.472 (4)	S3—O8	1.434 (4)
S1A—O2A	1.426 (4)	S3—O9	1.432 (4)
S1A—O3A	1.447 (4)	S3—C51	1.827 (6)
S1A—C49A	1.827 (6)	F7—C51	1.299 (8)
S1B—O2B	1.4453	F8—C51	1.338 (7)
S1B—O3B	1.4440	F9—C51	1.355 (8)
S1B—O1B	1.4452	S2—O4	1.436 (4)
S1B—C49B	1.8517	S2—O5	1.429 (4)
C49B—F3B	1.3392	S2—O6	1.424 (4)
C49B—F1B	1.3389	S2—C50	1.815 (6)
C49B—F2B	1.3388	F4—C50	1.341 (7)
F1A—C49A	1.328 (6)	F5—C50	1.320 (7)
F2A—C49A	1.341 (7)	F6—C50	1.343 (7)
			
O1—Zn1—N1	103.31 (14)	S1A—O1A—Zn1A	130.0 (3)
O1—Zn1—N2	107.79 (14)	C1A—N1A—Zn1A	132.5 (3)
O1—Zn1—N3	82.47 (14)	C13A—N1A—Zn1A	106.6 (3)
O1—Zn1—N4	105.35 (14)	C13A—N1A—C1A	118.2 (4)
N1—Zn1—N3	172.38 (14)	C14A—N2A—Zn1A	111.7 (3)
N2—Zn1—N1	80.55 (14)	C14A—N2A—C15A	120.6 (4)
N2—Zn1—N3	93.01 (14)	C15A—N2A—Zn1A	125.3 (3)
N2—Zn1—N4	143.22 (14)	Zn1A—N3A—H3A	105 (3)
N4—Zn1—N1	106.87 (14)	C34A—N3A—Zn1A	106.2 (3)
N4—Zn1—N3	75.90 (15)	C34A—N3A—H3A	111 (4)
O1—S1—C49	98.8 (2)	C34A—N3A—C35A	118.5 (4)
O2—S1—O1	112.5 (2)	C35A—N3A—Zn1A	100.5 (3)
O2—S1—C49	105.8 (2)	C35A—N3A—H3A	114 (4)
O3—S1—O1	112.5 (2)	C36A—N4A—Zn1A	107.1 (3)
O3—S1—O2	118.7 (2)	C36A—N4A—C48A	120.5 (4)
O3—S1—C49	106.1 (2)	C48A—N4A—Zn1A	126.8 (3)
S1—O1—Zn1	126.6 (2)	N1A—C1A—C2A	120.6 (4)
C1—N1—Zn1	130.5 (3)	N1A—C1A—C10A	120.5 (4)
C13—N1—Zn1	108.0 (3)	C10A—C1A—C2A	118.9 (4)
C13—N1—C1	117.9 (4)	C3A—C2A—C1A	123.1 (4)
C14—N2—Zn1	112.8 (3)	C3A—C2A—C7A	118.7 (5)
C14—N2—C15	120.9 (4)	C7A—C2A—C1A	118.1 (4)
C15—N2—Zn1	123.7 (3)	C2A—C3A—H3AA	119.3
Zn1—N3—H3	105 (4)	C4A—C3A—C2A	121.4 (5)
C34—N3—Zn1	103.6 (3)	C4A—C3A—H3AA	119.3
C34—N3—H3	112 (4)	C3A—C4A—H4A	120.1
C34—N3—C35	119.4 (4)	C3A—C4A—C5A	119.8 (5)
C35—N3—Zn1	101.5 (3)	C5A—C4A—H4A	120.1
C35—N3—H3	113 (4)	C4A—C5A—H5A	120.1
C36—N4—Zn1	108.8 (3)	C6A—C5A—C4A	119.7 (5)
C36—N4—C48	119.7 (4)	C6A—C5A—H5A	120.1
C48—N4—Zn1	125.4 (3)	C5A—C6A—H6A	119.2
N1—C1—C2	120.1 (4)	C5A—C6A—C7A	121.6 (5)
N1—C1—C10	120.8 (4)	C7A—C6A—H6A	119.2
C10—C1—C2	119.1 (4)	C2A—C7A—C8A	120.1 (5)
C3—C2—C1	122.6 (4)	C6A—C7A—C2A	118.6 (5)
C3—C2—C7	118.8 (4)	C6A—C7A—C8A	121.3 (5)
C7—C2—C1	118.6 (4)	C7A—C8A—H8A	119.3
C2—C3—H3B	119.4	C9A—C8A—C7A	121.3 (5)
C4—C3—C2	121.3 (4)	C9A—C8A—H8A	119.3
C4—C3—H3B	119.4	C8A—C9A—H9A	119.6
C3—C4—H4	119.9	C8A—C9A—C10A	120.8 (5)
C3—C4—C5	120.2 (5)	C10A—C9A—H9A	119.6
C5—C4—H4	119.9	C1A—C10A—C9A	119.9 (5)
C4—C5—H5	120.3	C11A—C10A—C1A	118.8 (5)
C6—C5—C4	119.4 (5)	C11A—C10A—C9A	121.3 (5)
C6—C5—H5	120.3	C10A—C11A—H11A	120.1
C5—C6—H6	119.2	C12A—C11A—C10A	119.8 (5)
C5—C6—C7	121.6 (4)	C12A—C11A—H11A	120.1
C7—C6—H6	119.2	C11A—C12A—H12A	120.6
C2—C7—C8	119.5 (4)	C11A—C12A—C13A	118.8 (5)
C6—C7—C2	118.6 (4)	C13A—C12A—H12A	120.6
C6—C7—C8	121.8 (4)	N1A—C13A—C12A	123.1 (5)
C7—C8—H8	119.1	N1A—C13A—C14A	117.6 (4)
C9—C8—C7	121.8 (4)	C12A—C13A—C14A	119.2 (5)
C9—C8—H8	119.1	N2A—C14A—C13A	120.4 (4)
C8—C9—H9	119.7	N2A—C14A—H14A	119.8
C8—C9—C10	120.6 (5)	C13A—C14A—H14A	119.8
C10—C9—H9	119.7	C16A—C15A—N2A	118.6 (4)
C1—C10—C9	119.4 (4)	C24A—C15A—N2A	120.3 (4)
C11—C10—C1	118.5 (4)	C24A—C15A—C16A	120.7 (4)
C11—C10—C9	122.1 (4)	C15A—C16A—H16A	119.8
C10—C11—H11	120.0	C17A—C16A—C15A	120.4 (5)
C12—C11—C10	120.0 (4)	C17A—C16A—H16A	119.8
C12—C11—H11	120.0	C16A—C17A—H17A	119.2
C11—C12—H12	121.1	C16A—C17A—C18A	121.6 (5)
C11—C12—C13	117.8 (4)	C18A—C17A—H17A	119.2
C13—C12—H12	121.1	C17A—C18A—C23A	118.2 (4)
N1—C13—C12	124.2 (4)	C19A—C18A—C17A	122.0 (4)
N1—C13—C14	115.7 (4)	C19A—C18A—C23A	119.7 (5)
C12—C13—C14	120.2 (4)	C18A—C19A—H19A	119.8
N2—C14—C13	119.6 (4)	C20A—C19A—C18A	120.5 (5)
N2—C14—H14	120.2	C20A—C19A—H19A	119.8
C13—C14—H14	120.2	C19A—C20A—H20A	120.0
C16—C15—N2	118.6 (4)	C19A—C20A—C21A	120.1 (5)
C24—C15—N2	119.3 (4)	C21A—C20A—H20A	120.0
C24—C15—C16	121.8 (4)	C20A—C21A—H21A	119.4
C15—C16—H16	120.0	C22A—C21A—C20A	121.2 (5)
C17—C16—C15	120.1 (4)	C22A—C21A—H21A	119.4
C17—C16—H16	120.0	C21A—C22A—H22A	119.5
C16—C17—H17	119.5	C21A—C22A—C23A	121.0 (5)
C16—C17—C18	121.1 (4)	C23A—C22A—H22A	119.5
C18—C17—H17	119.5	C18A—C23A—C24A	119.9 (4)
C17—C18—C19	121.9 (4)	C22A—C23A—C18A	117.5 (4)
C17—C18—C23	118.9 (4)	C22A—C23A—C24A	122.6 (4)
C19—C18—C23	119.1 (4)	C15A—C24A—C23A	119.1 (4)
C18—C19—H19	119.7	C15A—C24A—C25A	122.9 (4)
C20—C19—C18	120.7 (5)	C23A—C24A—C25A	117.9 (4)
C20—C19—H19	119.7	C26A—C25A—C24A	119.0 (4)
C19—C20—H20	119.7	C34A—C25A—C24A	121.8 (4)
C19—C20—C21	120.6 (4)	C34A—C25A—C26A	119.0 (4)
C21—C20—H20	119.7	C25A—C26A—C27A	121.7 (4)
C20—C21—H21	119.9	C31A—C26A—C25A	120.4 (4)
C22—C21—C20	120.1 (5)	C31A—C26A—C27A	117.9 (4)
C22—C21—H21	119.9	C26A—C27A—H27A	119.7
C21—C22—H22	119.4	C28A—C27A—C26A	120.6 (5)
C21—C22—C23	121.2 (5)	C28A—C27A—H27A	119.7
C23—C22—H22	119.4	C27A—C28A—H28A	119.4
C18—C23—C24	119.2 (4)	C27A—C28A—C29A	121.2 (5)
C22—C23—C18	118.2 (4)	C29A—C28A—H28A	119.4
C22—C23—C24	122.6 (4)	C28A—C29A—H29A	120.0
C15—C24—C23	118.8 (4)	C30A—C29A—C28A	120.0 (4)
C15—C24—C25	122.4 (4)	C30A—C29A—H29A	120.0
C23—C24—C25	118.7 (4)	C29A—C30A—H30A	119.7
C26—C25—C24	119.5 (4)	C29A—C30A—C31A	120.6 (5)
C34—C25—C24	120.5 (4)	C31A—C30A—H30A	119.7
C34—C25—C26	119.9 (4)	C26A—C31A—C30A	119.7 (4)
C27—C26—C25	122.7 (4)	C26A—C31A—C32A	118.3 (4)
C27—C26—C31	118.2 (4)	C30A—C31A—C32A	122.0 (4)
C31—C26—C25	119.1 (4)	C31A—C32A—H32A	119.4
C26—C27—H27	119.6	C33A—C32A—C31A	121.2 (4)
C28—C27—C26	120.9 (5)	C33A—C32A—H32A	119.4
C28—C27—H27	119.6	C32A—C33A—H33A	119.6
C27—C28—H28	119.8	C32A—C33A—C34A	120.7 (4)
C27—C28—C29	120.5 (5)	C34A—C33A—H33A	119.6
C29—C28—H28	119.8	C25A—C34A—N3A	118.7 (4)
C28—C29—H29	119.9	C25A—C34A—C33A	120.3 (4)
C30—C29—C28	120.2 (5)	C33A—C34A—N3A	121.0 (4)
C30—C29—H29	119.9	N3A—C35A—H35A	109.7
C29—C30—H30	119.7	N3A—C35A—H35B	109.7
C29—C30—C31	120.5 (5)	N3A—C35A—C36A	109.9 (4)
C31—C30—H30	119.7	H35A—C35A—H35B	108.2
C26—C31—C30	119.6 (5)	C36A—C35A—H35A	109.7
C26—C31—C32	118.9 (5)	C36A—C35A—H35B	109.7
C32—C31—C30	121.4 (5)	N4A—C36A—C35A	117.4 (4)
C31—C32—H32	119.5	N4A—C36A—C37A	122.0 (4)
C33—C32—C31	121.1 (5)	C37A—C36A—C35A	120.6 (4)
C33—C32—H32	119.5	C36A—C37A—H37A	120.8
C32—C33—H33	119.8	C38A—C37A—C36A	118.5 (5)
C32—C33—C34	120.4 (5)	C38A—C37A—H37A	120.8
C34—C33—H33	119.8	C37A—C38A—H38A	119.8
C25—C34—N3	118.4 (4)	C37A—C38A—C39A	120.4 (5)
C25—C34—C33	120.5 (4)	C39A—C38A—H38A	119.8
C33—C34—N3	120.9 (4)	C38A—C39A—C40A	122.0 (5)
N3—C35—H35C	109.5	C48A—C39A—C38A	118.3 (4)
N3—C35—H35D	109.5	C48A—C39A—C40A	119.7 (5)
N3—C35—C36	110.6 (4)	C39A—C40A—H40A	120.0
H35C—C35—H35D	108.1	C41A—C40A—C39A	120.0 (5)
C36—C35—H35C	109.5	C41A—C40A—H40A	120.0
C36—C35—H35D	109.5	C40A—C41A—H41A	119.0
N4—C36—C35	117.4 (4)	C40A—C41A—C42A	122.0 (5)
N4—C36—C37	121.7 (4)	C42A—C41A—H41A	119.0
C37—C36—C35	120.8 (4)	C41A—C42A—C47A	120.0 (5)
C36—C37—H37	120.6	C43A—C42A—C41A	121.5 (5)
C38—C37—C36	118.8 (5)	C43A—C42A—C47A	118.5 (5)
C38—C37—H37	120.6	C42A—C43A—H43A	119.4
C37—C38—H38	119.7	C44A—C43A—C42A	121.2 (5)
C37—C38—C39	120.7 (4)	C44A—C43A—H43A	119.4
C39—C38—H38	119.7	C43A—C44A—H44A	119.9
C38—C39—C40	123.2 (4)	C43A—C44A—C45A	120.3 (5)
C48—C39—C38	117.6 (4)	C45A—C44A—H44A	119.9
C48—C39—C40	119.2 (4)	C44A—C45A—H45A	119.9
C39—C40—H40	119.7	C46A—C45A—C44A	120.2 (5)
C41—C40—C39	120.7 (5)	C46A—C45A—H45A	119.9
C41—C40—H40	119.7	C45A—C46A—H46A	119.5
C40—C41—H41	119.4	C45A—C46A—C47A	121.1 (5)
C40—C41—C42	121.1 (5)	C47A—C46A—H46A	119.5
C42—C41—H41	119.4	C42A—C47A—C48A	117.3 (4)
C43—C42—C41	120.6 (5)	C46A—C47A—C42A	118.5 (5)
C47—C42—C41	119.9 (5)	C46A—C47A—C48A	124.1 (4)
C47—C42—C43	119.4 (5)	N4A—C48A—C39A	119.7 (4)
C42—C43—H43	120.0	N4A—C48A—C47A	119.8 (4)
C44—C43—C42	120.0 (5)	C39A—C48A—C47A	120.5 (4)
C44—C43—H43	120.0	F1A—C49A—S1A	111.8 (4)
C43—C44—H44	119.5	F1A—C49A—F2A	108.9 (4)
C43—C44—C45	121.1 (5)	F1A—C49A—F3A	106.5 (5)
C45—C44—H44	119.5	F2A—C49A—S1A	110.7 (4)
C44—C45—H45	120.1	F3A—C49A—S1A	111.4 (4)
C46—C45—C44	119.9 (5)	F3A—C49A—F2A	107.5 (5)
C46—C45—H45	120.1	Cl1—C52—H52A	109.6
C45—C46—H46	120.0	Cl1—C52—H52B	109.6
C45—C46—C47	120.1 (5)	Cl2—C52—Cl1	110.1 (3)
C47—C46—H46	120.0	Cl2—C52—H52A	109.6
C42—C47—C46	119.2 (4)	Cl2—C52—H52B	109.6
C42—C47—C48	118.2 (4)	H52A—C52—H52B	108.1
C46—C47—C48	122.4 (4)	Cl3—C53—H53A	109.3
N4—C48—C39	120.6 (4)	Cl3—C53—H53B	109.3
N4—C48—C47	119.4 (4)	Cl4—C53—Cl3	111.7 (4)
C39—C48—C47	120.0 (4)	Cl4—C53—H53A	109.3
F1—C49—S1	110.4 (3)	Cl4—C53—H53B	109.3
F1—C49—F2	107.8 (4)	H53A—C53—H53B	107.9
F1—C49—F3	108.3 (4)	Cl5—C54—Cl6	107 (2)
F2—C49—S1	110.7 (4)	Cl5—C54—H54A	110.3
F3—C49—S1	111.4 (3)	Cl5—C54—H54B	110.3
F3—C49—F2	108.2 (4)	Cl6—C54—H54A	110.3
O1B—Zn1A—N1A	89.7 (6)	Cl6—C54—H54B	110.3
O1B—Zn1A—N2A	100.0 (7)	H54A—C54—H54B	108.5
O1B—Zn1A—N3A	95.4 (6)	Cl5A—C54A—Cl6A	114 (2)
O1B—Zn1A—N4A	119.7 (7)	Cl5A—C54A—H54C	108.7
O1A—Zn1A—N1A	99.02 (19)	Cl5A—C54A—H54D	108.7
O1A—Zn1A—N3A	86.49 (18)	Cl6A—C54A—H54C	108.7
O1A—Zn1A—N4A	109.2 (2)	Cl6A—C54A—H54D	108.7
N1A—Zn1A—N3A	174.03 (14)	H54C—C54A—H54D	107.6
N2A—Zn1A—O1A	109.05 (19)	O7—S3—O8	114.1 (2)
N2A—Zn1A—N1A	82.08 (15)	O7—S3—O9	116.6 (3)
N2A—Zn1A—N3A	94.00 (15)	O7—S3—C51	103.0 (3)
N2A—Zn1A—N4A	139.56 (14)	O8—S3—C51	102.5 (3)
N4A—Zn1A—N1A	104.21 (15)	O9—S3—O8	114.8 (3)
N4A—Zn1A—N3A	75.87 (15)	O9—S3—C51	103.1 (3)
O1A—S1A—C49A	100.7 (3)	F7—C51—S3	113.4 (5)
O2A—S1A—O1A	113.5 (3)	F7—C51—F8	107.5 (5)
O2A—S1A—O3A	117.6 (2)	F7—C51—F9	107.4 (5)
O2A—S1A—C49A	106.1 (2)	F8—C51—S3	110.5 (4)
O3A—S1A—O1A	112.3 (2)	F8—C51—F9	107.6 (5)
O3A—S1A—C49A	104.5 (2)	F9—C51—S3	110.2 (4)
O2B—S1B—C49B	102.3	O4—S2—C50	103.3 (3)
O3B—S1B—O2B	115.5	O5—S2—O4	115.2 (3)
O3B—S1B—O1B	115.6	O5—S2—C50	102.5 (3)
O3B—S1B—C49B	102.4	O6—S2—O4	115.5 (3)
O1B—S1B—O2B	115.5	O6—S2—O5	115.3 (3)
O1B—S1B—C49B	102.4	O6—S2—C50	102.2 (3)
S1B—O1B—Zn1A	133.2 (14)	F4—C50—S2	110.8 (4)
F3B—C49B—S1B	111.8	F4—C50—F6	106.5 (5)
F1B—C49B—S1B	111.9	F5—C50—S2	112.9 (4)
F1B—C49B—F3B	106.9	F5—C50—F4	106.7 (5)
F2B—C49B—S1B	111.9	F5—C50—F6	108.2 (5)
F2B—C49B—F3B	106.9	F6—C50—S2	111.4 (4)
F2B—C49B—F1B	107.0		
			
Zn1—N1—C1—C2	31.6 (6)	O2B—S1B—C49B—F3B	180.0
Zn1—N1—C1—C10	−147.7 (3)	O2B—S1B—C49B—F1B	−60.0
Zn1—N1—C13—C12	160.4 (4)	O2B—S1B—C49B—F2B	60.0
Zn1—N1—C13—C14	−19.4 (4)	O3B—S1B—O1B—Zn1A	133.3 (16)
Zn1—N2—C14—C13	−7.6 (5)	O3B—S1B—C49B—F3B	60.0
Zn1—N2—C15—C16	−116.0 (4)	O3B—S1B—C49B—F1B	−180.0
Zn1—N2—C15—C24	58.6 (5)	O3B—S1B—C49B—F2B	−59.9
Zn1—N3—C34—C25	76.6 (4)	O1B—S1B—C49B—F3B	−60.0
Zn1—N3—C34—C33	−98.4 (4)	O1B—S1B—C49B—F1B	60.0
Zn1—N3—C35—C36	35.4 (4)	O1B—S1B—C49B—F2B	−180.0
Zn1—N4—C36—C35	−34.5 (5)	C49B—S1B—O1B—Zn1A	−116.3 (16)
Zn1—N4—C36—C37	146.0 (4)	O1A—S1A—C49A—F1A	63.5 (4)
Zn1—N4—C48—C39	−137.9 (4)	O1A—S1A—C49A—F2A	−175.0 (4)
Zn1—N4—C48—C47	44.4 (5)	O1A—S1A—C49A—F3A	−55.5 (5)
O1—S1—C49—F1	58.8 (4)	O2A—S1A—O1A—Zn1A	−155.7 (4)
O1—S1—C49—F2	178.1 (3)	O2A—S1A—C49A—F1A	−55.0 (4)
O1—S1—C49—F3	−61.5 (4)	O2A—S1A—C49A—F2A	66.5 (4)
O2—S1—O1—Zn1	−80.9 (3)	O2A—S1A—C49A—F3A	−174.0 (5)
O2—S1—C49—F1	−57.7 (4)	O3A—S1A—O1A—Zn1A	−19.3 (6)
O2—S1—C49—F2	61.6 (4)	O3A—S1A—C49A—F1A	−180.0 (4)
O2—S1—C49—F3	−178.0 (3)	O3A—S1A—C49A—F2A	−58.4 (4)
O3—S1—O1—Zn1	56.3 (3)	O3A—S1A—C49A—F3A	61.0 (5)
O3—S1—C49—F1	175.3 (3)	N1A—C1A—C2A—C3A	14.3 (7)
O3—S1—C49—F2	−65.4 (4)	N1A—C1A—C2A—C7A	−167.9 (4)
O3—S1—C49—F3	55.0 (4)	N1A—C1A—C10A—C9A	169.7 (4)
N1—C1—C2—C3	14.0 (6)	N1A—C1A—C10A—C11A	−9.9 (7)
N1—C1—C2—C7	−168.9 (4)	N1A—C13A—C14A—N2A	14.8 (7)
N1—C1—C10—C9	169.6 (4)	N2A—C15A—C16A—C17A	171.9 (4)
N1—C1—C10—C11	−9.0 (6)	N2A—C15A—C24A—C23A	−172.0 (4)
N1—C13—C14—N2	19.6 (6)	N2A—C15A—C24A—C25A	10.2 (7)
N2—C15—C16—C17	171.6 (4)	N3A—C35A—C36A—N4A	−6.4 (6)
N2—C15—C24—C23	−172.0 (4)	N3A—C35A—C36A—C37A	171.1 (4)
N2—C15—C24—C25	9.3 (6)	N4A—C36A—C37A—C38A	−0.6 (7)
N3—C35—C36—N4	−4.0 (6)	C1A—N1A—C13A—C12A	1.4 (7)
N3—C35—C36—C37	175.4 (4)	C1A—N1A—C13A—C14A	−176.6 (4)
N4—C36—C37—C38	−0.2 (7)	C1A—C2A—C3A—C4A	−178.6 (4)
C1—N1—C13—C12	−0.6 (6)	C1A—C2A—C7A—C6A	177.6 (4)
C1—N1—C13—C14	179.6 (4)	C1A—C2A—C7A—C8A	−4.7 (7)
C1—C2—C3—C4	−178.4 (4)	C1A—C10A—C11A—C12A	4.2 (8)
C1—C2—C7—C6	178.3 (4)	C2A—C1A—C10A—C9A	−8.6 (7)
C1—C2—C7—C8	−3.7 (6)	C2A—C1A—C10A—C11A	171.8 (4)
C1—C10—C11—C12	2.0 (7)	C2A—C3A—C4A—C5A	−0.2 (7)
C2—C1—C10—C9	−9.7 (6)	C2A—C7A—C8A—C9A	−3.3 (8)
C2—C1—C10—C11	171.7 (4)	C3A—C2A—C7A—C6A	−4.4 (7)
C2—C3—C4—C5	−1.2 (8)	C3A—C2A—C7A—C8A	173.2 (4)
C2—C7—C8—C9	−4.2 (7)	C3A—C4A—C5A—C6A	−2.3 (8)
C3—C2—C7—C6	−4.5 (7)	C4A—C5A—C6A—C7A	1.4 (8)
C3—C2—C7—C8	173.5 (4)	C5A—C6A—C7A—C2A	2.0 (8)
C3—C4—C5—C6	−2.2 (8)	C5A—C6A—C7A—C8A	−175.6 (5)
C4—C5—C6—C7	2.2 (8)	C6A—C7A—C8A—C9A	174.4 (5)
C5—C6—C7—C2	1.2 (7)	C7A—C2A—C3A—C4A	3.6 (7)
C5—C6—C7—C8	−176.8 (5)	C7A—C8A—C9A—C10A	5.4 (9)
C6—C7—C8—C9	173.7 (5)	C8A—C9A—C10A—C1A	0.6 (8)
C7—C2—C3—C4	4.5 (7)	C8A—C9A—C10A—C11A	−179.8 (5)
C7—C8—C9—C10	5.1 (8)	C9A—C10A—C11A—C12A	−175.3 (5)
C8—C9—C10—C1	1.9 (7)	C10A—C1A—C2A—C3A	−167.4 (4)
C8—C9—C10—C11	−179.5 (5)	C10A—C1A—C2A—C7A	10.4 (6)
C9—C10—C11—C12	−176.6 (4)	C10A—C11A—C12A—C13A	3.8 (8)
C10—C1—C2—C3	−166.7 (4)	C11A—C12A—C13A—N1A	−6.9 (8)
C10—C1—C2—C7	10.4 (6)	C11A—C12A—C13A—C14A	171.0 (5)
C10—C11—C12—C13	5.2 (7)	C12A—C13A—C14A—N2A	−163.2 (4)
C11—C12—C13—N1	−6.2 (7)	C13A—N1A—C1A—C2A	−174.7 (4)
C11—C12—C13—C14	173.6 (4)	C13A—N1A—C1A—C10A	7.0 (6)
C12—C13—C14—N2	−160.2 (4)	C14A—N2A—C15A—C16A	42.5 (6)
C13—N1—C1—C2	−172.5 (4)	C14A—N2A—C15A—C24A	−144.0 (4)
C13—N1—C1—C10	8.2 (6)	C15A—N2A—C14A—C13A	−170.4 (4)
C14—N2—C15—C16	44.5 (6)	C15A—C16A—C17A—C18A	0.6 (7)
C14—N2—C15—C24	−140.8 (4)	C15A—C24A—C25A—C26A	108.6 (5)
C15—N2—C14—C13	−170.1 (4)	C15A—C24A—C25A—C34A	−76.5 (6)
C15—C16—C17—C18	1.3 (7)	C16A—C15A—C24A—C23A	1.2 (7)
C15—C24—C25—C26	104.5 (5)	C16A—C15A—C24A—C25A	−176.5 (4)
C15—C24—C25—C34	−77.8 (6)	C16A—C17A—C18A—C19A	−178.9 (4)
C16—C15—C24—C23	2.4 (6)	C16A—C17A—C18A—C23A	0.4 (7)
C16—C15—C24—C25	−176.2 (4)	C17A—C18A—C19A—C20A	179.9 (5)
C16—C17—C18—C19	−178.3 (4)	C17A—C18A—C23A—C22A	−179.5 (4)
C16—C17—C18—C23	0.6 (7)	C17A—C18A—C23A—C24A	−0.7 (7)
C17—C18—C19—C20	−179.6 (4)	C18A—C19A—C20A—C21A	−0.6 (8)
C17—C18—C23—C22	−179.2 (4)	C18A—C23A—C24A—C15A	−0.2 (7)
C17—C18—C23—C24	−1.0 (6)	C18A—C23A—C24A—C25A	177.7 (4)
C18—C19—C20—C21	−2.1 (7)	C19A—C18A—C23A—C22A	−0.1 (7)
C18—C23—C24—C15	−0.5 (6)	C19A—C18A—C23A—C24A	178.7 (4)
C18—C23—C24—C25	178.3 (4)	C19A—C20A—C21A—C22A	0.4 (8)
C19—C18—C23—C22	−0.2 (6)	C20A—C21A—C22A—C23A	0.0 (8)
C19—C18—C23—C24	177.9 (4)	C21A—C22A—C23A—C18A	−0.2 (7)
C19—C20—C21—C22	1.5 (7)	C21A—C22A—C23A—C24A	−178.9 (4)
C20—C21—C22—C23	−0.3 (7)	C22A—C23A—C24A—C15A	178.6 (4)
C21—C22—C23—C18	−0.3 (7)	C22A—C23A—C24A—C25A	−3.6 (6)
C21—C22—C23—C24	−178.4 (4)	C23A—C18A—C19A—C20A	0.5 (7)
C22—C23—C24—C15	177.6 (4)	C23A—C24A—C25A—C26A	−69.2 (5)
C22—C23—C24—C25	−3.7 (6)	C23A—C24A—C25A—C34A	105.8 (5)
C23—C18—C19—C20	1.4 (7)	C24A—C15A—C16A—C17A	−1.5 (7)
C23—C24—C25—C26	−74.2 (5)	C24A—C25A—C26A—C27A	−8.4 (6)
C23—C24—C25—C34	103.5 (5)	C24A—C25A—C26A—C31A	171.5 (4)
C24—C15—C16—C17	−2.9 (7)	C24A—C25A—C34A—N3A	10.4 (6)
C24—C25—C26—C27	−6.4 (6)	C24A—C25A—C34A—C33A	−172.2 (4)
C24—C25—C26—C31	174.5 (4)	C25A—C26A—C27A—C28A	179.2 (4)
C24—C25—C34—N3	8.6 (6)	C25A—C26A—C31A—C30A	−177.8 (4)
C24—C25—C34—C33	−176.4 (4)	C25A—C26A—C31A—C32A	1.8 (6)
C25—C26—C27—C28	−179.8 (4)	C26A—C25A—C34A—N3A	−174.6 (4)
C25—C26—C31—C30	−178.7 (4)	C26A—C25A—C34A—C33A	2.7 (6)
C25—C26—C31—C32	2.7 (6)	C26A—C27A—C28A—C29A	−1.4 (7)
C26—C25—C34—N3	−173.7 (4)	C26A—C31A—C32A—C33A	0.8 (7)
C26—C25—C34—C33	1.3 (6)	C27A—C26A—C31A—C30A	2.1 (6)
C26—C27—C28—C29	−2.1 (7)	C27A—C26A—C31A—C32A	−178.3 (4)
C26—C31—C32—C33	−0.4 (7)	C27A—C28A—C29A—C30A	2.1 (7)
C27—C26—C31—C30	2.2 (6)	C28A—C29A—C30A—C31A	−0.6 (7)
C27—C26—C31—C32	−176.4 (4)	C29A—C30A—C31A—C26A	−1.5 (7)
C27—C28—C29—C30	3.4 (7)	C29A—C30A—C31A—C32A	178.9 (4)
C28—C29—C30—C31	−1.8 (7)	C30A—C31A—C32A—C33A	−179.5 (4)
C29—C30—C31—C26	−1.0 (7)	C31A—C26A—C27A—C28A	−0.7 (6)
C29—C30—C31—C32	177.6 (5)	C31A—C32A—C33A—C34A	−1.7 (7)
C30—C31—C32—C33	−179.0 (4)	C32A—C33A—C34A—N3A	177.2 (4)
C31—C26—C27—C28	−0.7 (6)	C32A—C33A—C34A—C25A	−0.1 (6)
C31—C32—C33—C34	−1.5 (7)	C34A—N3A—C35A—C36A	−75.9 (5)
C32—C33—C34—N3	176.0 (4)	C34A—C25A—C26A—C27A	176.5 (4)
C32—C33—C34—C25	1.1 (7)	C34A—C25A—C26A—C31A	−3.6 (6)
C34—N3—C35—C36	−77.6 (5)	C35A—N3A—C34A—C25A	−174.4 (4)
C34—C25—C26—C27	175.9 (4)	C35A—N3A—C34A—C33A	8.3 (6)
C34—C25—C26—C31	−3.2 (6)	C35A—C36A—C37A—C38A	−178.0 (4)
C35—N3—C34—C25	−171.6 (4)	C36A—N4A—C48A—C39A	10.2 (6)
C35—N3—C34—C33	13.5 (6)	C36A—N4A—C48A—C47A	−169.0 (4)
C35—C36—C37—C38	−179.6 (4)	C36A—C37A—C38A—C39A	4.7 (7)
C36—N4—C48—C39	11.2 (6)	C37A—C38A—C39A—C40A	179.1 (5)
C36—N4—C48—C47	−166.5 (4)	C37A—C38A—C39A—C48A	−1.4 (7)
C36—C37—C38—C39	4.7 (7)	C38A—C39A—C40A—C41A	−179.4 (5)
C37—C38—C39—C40	179.4 (5)	C38A—C39A—C48A—N4A	−6.0 (7)
C37—C38—C39—C48	−1.3 (7)	C38A—C39A—C48A—C47A	173.2 (4)
C38—C39—C40—C41	−178.6 (5)	C39A—C40A—C41A—C42A	3.6 (8)
C38—C39—C48—N4	−6.7 (6)	C40A—C39A—C48A—N4A	173.5 (4)
C38—C39—C48—C47	171.0 (4)	C40A—C39A—C48A—C47A	−7.3 (7)
C39—C40—C41—C42	4.2 (7)	C40A—C41A—C42A—C43A	176.9 (5)
C40—C39—C48—N4	172.7 (4)	C40A—C41A—C42A—C47A	−2.2 (8)
C40—C39—C48—C47	−9.6 (6)	C41A—C42A—C43A—C44A	−175.9 (5)
C40—C41—C42—C43	174.4 (5)	C41A—C42A—C47A—C46A	173.3 (5)
C40—C41—C42—C47	−2.8 (7)	C41A—C42A—C47A—C48A	−3.9 (7)
C41—C42—C43—C44	−174.7 (5)	C42A—C43A—C44A—C45A	0.5 (9)
C41—C42—C47—C46	171.6 (4)	C42A—C47A—C48A—N4A	−172.3 (4)
C41—C42—C47—C48	−4.7 (7)	C42A—C47A—C48A—C39A	8.5 (6)
C42—C43—C44—C45	0.9 (8)	C43A—C42A—C47A—C46A	−5.8 (7)
C42—C47—C48—N4	−171.4 (4)	C43A—C42A—C47A—C48A	177.0 (4)
C42—C47—C48—C39	10.8 (6)	C43A—C44A—C45A—C46A	−1.6 (8)
C43—C42—C47—C46	−5.7 (7)	C44A—C45A—C46A—C47A	−1.2 (8)
C43—C42—C47—C48	178.1 (4)	C45A—C46A—C47A—C42A	4.8 (7)
C43—C44—C45—C46	−1.1 (8)	C45A—C46A—C47A—C48A	−178.2 (4)
C44—C45—C46—C47	−2.1 (7)	C46A—C47A—C48A—N4A	10.7 (7)
C45—C46—C47—C42	5.5 (7)	C46A—C47A—C48A—C39A	−168.5 (4)
C45—C46—C47—C48	−178.4 (4)	C47A—C42A—C43A—C44A	3.2 (8)
C46—C47—C48—N4	12.4 (7)	C48A—N4A—C36A—C35A	170.6 (4)
C46—C47—C48—C39	−165.3 (4)	C48A—N4A—C36A—C37A	−6.9 (6)
C47—C42—C43—C44	2.5 (8)	C48A—C39A—C40A—C41A	1.1 (7)
C48—N4—C36—C35	171.7 (4)	C49A—S1A—O1A—Zn1A	91.3 (5)
C48—N4—C36—C37	−7.7 (7)	O7—S3—C51—F7	−63.0 (5)
C48—C39—C40—C41	2.1 (7)	O7—S3—C51—F8	57.8 (5)
C49—S1—O1—Zn1	167.8 (3)	O7—S3—C51—F9	176.6 (4)
Zn1A—N1A—C1A—C2A	26.9 (6)	O8—S3—C51—F7	178.3 (5)
Zn1A—N1A—C1A—C10A	−151.3 (4)	O8—S3—C51—F8	−60.9 (5)
Zn1A—N1A—C13A—C12A	164.9 (4)	O8—S3—C51—F9	57.9 (5)
Zn1A—N1A—C13A—C14A	−13.1 (5)	O9—S3—C51—F7	58.7 (5)
Zn1A—N2A—C14A—C13A	−7.1 (5)	O9—S3—C51—F8	179.5 (5)
Zn1A—N2A—C15A—C16A	−118.4 (4)	O9—S3—C51—F9	−61.6 (5)
Zn1A—N2A—C15A—C24A	55.0 (5)	O4—S2—C50—F4	−61.3 (5)
Zn1A—N3A—C34A—C25A	73.7 (4)	O4—S2—C50—F5	58.4 (5)
Zn1A—N3A—C34A—C33A	−103.6 (4)	O4—S2—C50—F6	−179.6 (4)
Zn1A—N3A—C35A—C36A	39.2 (4)	O5—S2—C50—F4	178.7 (4)
Zn1A—N4A—C36A—C35A	−34.1 (4)	O5—S2—C50—F5	−61.7 (5)
Zn1A—N4A—C36A—C37A	148.4 (4)	O5—S2—C50—F6	60.3 (5)
Zn1A—N4A—C48A—C39A	−139.9 (4)	O6—S2—C50—F4	59.0 (5)
Zn1A—N4A—C48A—C47A	40.9 (5)	O6—S2—C50—F5	178.6 (4)
O2B—S1B—O1B—Zn1A	−6.0 (16)	O6—S2—C50—F6	−59.3 (5)
